# The Role of Stretching-Induced Phase Transformations in the Mechanical Properties of Isotactic 1‑Butene-ethylene Copolymers from Ziegler–Natta Catalyst

**DOI:** 10.1021/acs.macromol.5c01958

**Published:** 2025-12-11

**Authors:** Anna Malafronte, Rocco Di Girolamo, Angelo Giordano, Fabio De Stefano, Miriam Scoti, Claudio De Rosa

**Affiliations:** Dipartimento di Scienze Chimiche, Università di Napoli Federico II, Complesso Monte S.Angelo, Via Cintia, 80126 Napoli, Italy

## Abstract

The crystallization behavior and phase transformations occurring during deformation of random isotactic 1-butene-ethylene (C4C2) copolymers, synthesized with a Ziegler–Natta catalyst and characterized by an ethylene (C2) content between 1.7 and 16.3 mol %, were investigated using a combination of tensile testing and *in situ* wide-angle X-ray diffraction (WAXD) with synchrotron radiation. The phase transformations were correlated to the mechanical behavior. Samples with C2 content ≤7.6 mol % were basically crystallized in form II in their unstretched state, whereas samples with higher C2 contents were crystallized as a mixture of form II and form I′. During stretching, form II present in all the initial unoriented samples rapidly transforms into form I, indicating that uniaxial deformation significantly accelerates form II–form I transition, since in quiescent conditions, this transformation required longer times to complete. However, the effect of stretching on the form II–form I transition differs among the various copolymers. Specifically, the critical strain at which the form II–form I transition begins (ε_c_) and the strain at which 50% of the initial form II transforms into form I (ε_0.5_) increase with increasing C2 content. The quantitative analysis of WAXD data enabled the construction of a phase diagram for C4C2 copolymers, which identified the regions of stability of the various polymorphic forms as a function of ethylene content and deformation. In addition to the form II–form I transition, further crystallization of form I′ from the amorphous phase was observed during stretching of some samples. This study provides valuable structure–property information that is helpful for the design and application of this class of semicrystalline materials.

## Graphical abstract

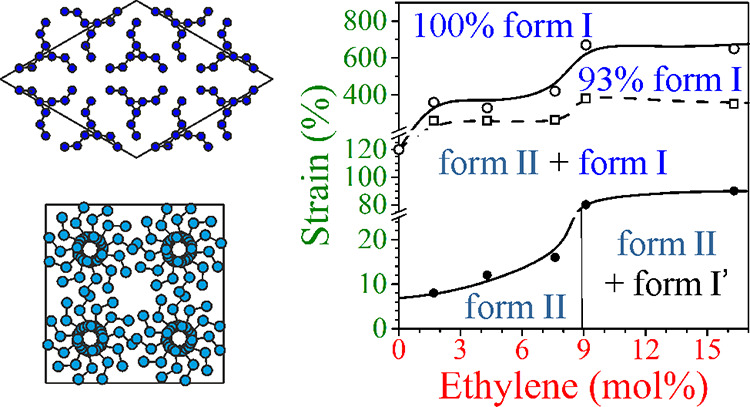

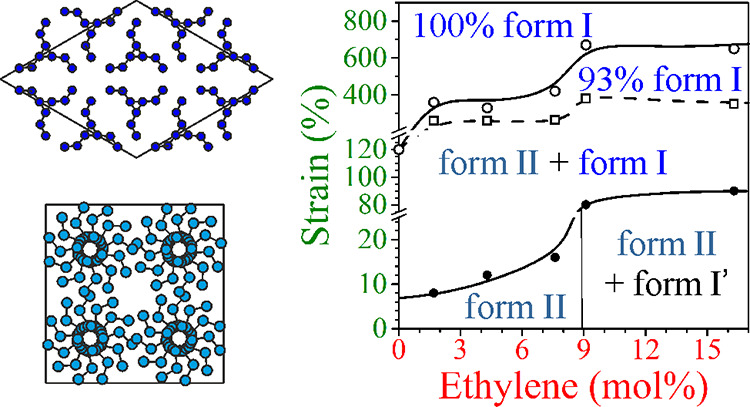

## Introduction

The investigation of deformation mechanisms in semicrystalline polymers, and how mechanical properties are related to microstructure, morphology, crystallinity, and molecular mass of polymers and copolymers, is of great scientific and industrial interest.^1–25^ However, because of the complex, hierarchical organization of polymer morphology, it remains difficult to draw definitive connections between their overall mechanical behavior and the microscopic structural features present at different length scales.^1–9^ These studies are further complicated in the case of polymorphic semicrystalline polymers because, during tensile or other types of mechanical deformation, external force may induce phase transitions that affect the stress–strain curve and, consequently, the mechanical behavior.^10,11,14–16,18,20,22,25–31^ Such mechanically induced phase transformations are particularly relevant from an application standpoint for isotactic poly­(1-butene) (iPB).^32–42^ iPB crystallizes in three different polymorphic forms, known as forms I, II, and III, which contain chains in different conformations and distinct crystal packing arrangements.^18,31,43–46^ iPB produced using traditional Ziegler–Natta (ZN) catalysts typically crystallizes from the melt into form II. This metastable form transforms into the more stable form I over time by aging at ambient temperature.^43,47–50^ The spontaneous transformation of form II into form I at ambient temperature leads to changes in physical properties during aging, such as an increase of rigidity and strength, as well as higher density and melting temperature.^22,43,47–49^ The form II → form I transformation is rather slow, requiring several days or even weeks at ambient temperature to complete.^43,47–49^ The sluggish nature of this phase transition increases manufacturing expenses and has limited the widespread commercial adoption of iPB. Since the superior mechanical performance of poly­(1-butene) is largely linked to the presence of form I, considerable research has been aimed to speed up the transition or to discover conditions that enable the direct crystallization of form I (termed form I′). It is important to remember that form I′ is a structurally imperfect variant of form I that crystallizes directly from the melt or amorphous phase, rather than being produced through the conversion of form II.^18,22,31,51^ One effective strategy for promoting the direct crystallization of form I′ and speeding up the transformation from form II to form I is to modify the molecular architecture of the iPB chains by introducing ethylene comonomer units.^21,31,52–62^ The ethylene (C2) units diminish the length of regular sequences of butene, significantly enhancing the kinetics of the form II-to-form I transition in 1-butene–ethylene (C4C2) copolymers compared to the poly­(1-butene) homopolymer and encouraging the direct crystallization of form I′.^52–62^

The form II–form I transition is also sped up by the application of external mechanical stress,^33–42^ which introduces forces—either internal or external—that promote the nucleation of form I crystals within the early lamellar structures of form II. Studies on transformation triggered by stretching in iPB homopolymer^33–42^ have revealed that the phase transition initiates once a critical stress level is reached, a value that consistently matches the tensile yield stress.^35–37,41,42,63,64^ As the strain continues beyond the yield point, the transition proceeds much more rapidly until completion. At this stage, form I becomes the dominant phase, and the stress–strain curve clearly shows strain hardening behavior.^35–37,42^ Liu et al.^35^ categorized the transition process into three distinct phases: incubation, nucleation, and the development (gelation) of form I crystals. These phases align with the regions of linear deformation, the stress plateau, and the strain hardening phase on the engineering stress–strain curve, respectively. The strain hardening is attributed to the emergence of a new mechanical network, where form I crystals serve as physical cross-linking points (gelation). Alternatively, Cavallo et al.^37^ interpreted the strain hardening as a result of the replacement of form II crystals by form I, which demands a significantly higher stress to maintain plastic deformation. Their findings indicated that the phase change was primarily driven by stress, until the fraction of form I reached about 50%. The slower transformation kinetics observed in the later stages were linked to the formation of a mechanical network made of form I crystals, which then carried most of the applied stress load.

The tensile-induced crystal–crystal transition in C4C2 copolymers has been less extensively explored.^57,65,66^ Although the accelerating effect of stress and strain on the form II–form I transition has been confirmed, detailed quantitative correlations between the degree of phase transition and mechanical response are rarely reported. To the best of our knowledge, existing studies are limited to a copolymer synthesized by Ziegler–Natta catalysis containing a very low amount of ethylene counits (1.5 mol %)^65,66^ and thus exhibiting a crystallization behavior similar to that of the iPB homopolymer. Three orientation pathways, depending on stretching conditions (drawing rate and temperature), were identified for the stretching-induced II–I phase transition in the studied C4C2 copolymer: (i) phase transition occurring within off-axis oriented crystallites, (ii) phase transition accompanied by the simultaneous formation of highly oriented crystallites, and (iii) phase transition taking place within already formed highly oriented crystallites.^65^ Wang et al.^66^ also prepared II/I mixture systems of the same C4C2 copolymer with different fractions of form I (by exploiting the quiescent phase transition from form II into form I) and investigated the stretching-induced phase transition in the mixture systems. They observed that the onset of the stretching-induced further transition from form II to form I always coincides with the mechanical yield point of the system, as also occurs in the case of the iPB homopolymer. It was hypothesized that at the yield strain, tie chains develop from the original folded chains and loops, providing nucleation sites for form I formation. Moreover, for all II/I mixture systems, the threshold stresses required for triggering the phase transition, i.e., the yield stress, followed the master curve correlating form I fraction with stress obtained from the pure initial form II system, confirming the stress-governed kinetics of the phase transition.

Understanding the structural changes that occur at the microscopic level during deformation of compression-molded films of C4C2 copolymers is essential for fully interpreting the mechanical stress–strain behavior and tailoring the mechanical performance of these polymeric materials. Since structural evolution and mechanical properties are dynamically coupled during deformation, in situ measurements combining structure and mechanical characterization techniques are crucial to elucidate structure–property relations.^35,37,42,64–66^

In this work, we present a quantitative analysis of the structural transformations and crystallization occurring during tensile deformation of random C4C2 copolymers synthesized with a ZN catalyst in a wide range of C2 content, by combining an in situ tensile tester with synchrotron radiation wide-angle X-ray diffraction techniques. Analysis of diffraction patterns recorded during deformation enabled the construction of an informative phase diagram for C4C2 copolymers, which identifies the regions of stability of the various polymorphic forms as a function of the ethylene content and deformation.

## Experimental Section

### Materials

A series of random isotactic 1-butene-ethylene (C4C2) copolymers containing varying amounts of ethylene, ranging from 1.7 to 16.3 mol %, were synthesized using a Ziegler–Natta catalytic system based on a MgCl_2_ support. The synthesis involved di­(isobutyl) phthalate as the internal electron donor, tri-isobutylaluminum (TIBA) as the cocatalyst, and (2,3-dimethyl-butan-2-yl)­trimethoxysilane (thexyltrimethoxysilane) as the external electron donor.^58^ All samples were characterized by ^13^C NMR spectroscopy (Figure S1). The ^13^C NMR spectrum of the iPB homopolymer, synthesized under the same conditions as those for the C4C2 copolymers, is also included in Figure S1 for comparison. Details of the studied samples are listed in Table 1. The copolymers are labeled as C4C2-X, where X denotes the ethylene (C2) content in mol % within each sample. C2 content, as well as the average lengths of ethylene and butene sequences (*n*(E) and *n*(B)), were estimated from ^13^C NMR spectra using the procedure described in Supporting Information (Table S1). Samples C4C2–16.3 and C4C2–7.6 are fractions derived from original sample C4C2–9.1 through Kumagawa extraction, utilizing sequential boiling with diethyl ether (EE) and hexane (HE) as solvents. More specifically, C4C2–16.3 corresponds to the fraction soluble in EE, whereas C4C2–7.6 represents the portion that is insoluble in EE but dissolves in HE.^58^ The iPB homopolymer, synthesized with the same catalyst and under the same conditions used for the C4C2 copolymers, shows a content of fully isotactic *mmmm* pentads of 97.5% (Figure S2),^67,68^ which is likely retained in the copolymers (Figure S3).

**Table 1:** Ethylene (C2) Content, Intrinsic Viscosities [η], Average Molecular Masses (*M*_v_ and *M*_w_), Polydispersity Indexes (*M*_w_/*M*_n_), and Average Lengths of Ethylene and Butene Sequences (*n*(E) and *n*(B)) for Random Isotactic 1-Butene-Ethylene (C4C2) Copolymers Analyzed in This Work

sample	C2 (mol %)^a^	[η] (dL/g)^b^	*M*_v_ × 10^–3^ (g/mol)^c^	*M*_*w*_ × 10^–3^ (g/mol)^d^	*M*_w_/*M*_n_ ^d^	*n*(E)^a^	*n*(B)^a^
C4C2–1.7	1.7	1.93	367	418	4.9	1.0	24.6
C4C2–4.3	4.3	1.50	260	297	3.9	1.01	22.00
C4C2–5.5	5.5	1.45	248	283	3.9	1.05	15.75
C4C2–7.6	7.6	2.23	418	464	2.7	1.12	12.85
C4C2–9.1	9.1	1.97	378	427	3.0	1.14	11.07
C4C2–14.3	14.3	1.58	279	315	3.1	1.17	6.92
C4C2–16.3	16.3	0.56	57.8	67.9	6.2	1.19	5.91

a From ^13^C NMR analysis (see Supporting Information).^58^

b Measured in tetrahydronaphthalene at 135 °C using standard Ubbelohde viscometer.

c From the values of intrinsic viscosity.^58^

d From GPC.

### In Situ X-ray Diffraction during Tensile Deformation

X-ray diffraction measurements during tensile deformation were conducted with synchrotron radiation at the beamline BM26-DUBBLE at ESRF.^69,70^ Unoriented compression-molded films (thickness 0.1–0.35 mm) were prepared by melting samples at temperatures 30–40 °C higher than their melting temperature for 2–3 min under a pressure lower than 5 bar, to avoid preferred orientations in the film, and cooling to ambient temperature by air quenching. Rectangular specimens (4.6–5.4 mm width) cut from unoriented compression-molded films were stretched at ambient temperature at a drawing rate equal to 333 μm/s. The sample clamps were properly aligned such that the wider surface of the sample was oriented perpendicular to the X-ray beam. Synchrotron X-ray radiation with a wavelength of 1.033 Å was used, focused on the sample with a spot size of approximately 400 μm x 300 μm. Two-dimensional (2D) wide-angle X-ray diffraction (WAXD) patterns were collected during deformation in transmission mode by using a Pilatus 1 M detector, positioned at a sample-to-detector distance of about 173 mm. For each WAXD frame, the acquisition period including exposure and recording steps was 2 s. The angular scale and thus the module of the scattering vector *q* scale (*q* = 4π sin θ/λ) were calibrated using α-Al_2_O_3_ standard. The 2D WAXD patterns were azimuthally averaged and radially integrated, resulting in one-dimensional (1D) diffraction profiles of scattered intensity as a function of *q*. The degree of crystallinity (*x*_c_) and the content of different crystalline phases were evaluated from 1D diffraction patterns by using the procedure described in the Supporting Information.

## Results and Discussion

### Crystallization Behavior of Unoriented Samples and Mechanical Properties

X-ray powder diffraction profiles of unoriented, compression-molded films of random C4C2 copolymers recorded immediately after preparation (referred to as fresh samples) and after prolonged aging at ambient temperature (7 days or more; referred to as aged samples) are presented in Figure 1A,B, respectively. The degrees of crystallinity (*x*_c_) for both fresh and aged samples, along with the amount of the various crystalline phases, are summarized in Table S2. We recall that form I of iPB crystallizes with two different modifications, showing the same diffraction profile. One is termed form I, which results from the transformation of form II, and the other is the low-melting form I′, which forms either by direct crystallization from the melt or by crystallization from the amorphous phase.

**Figure 1:**
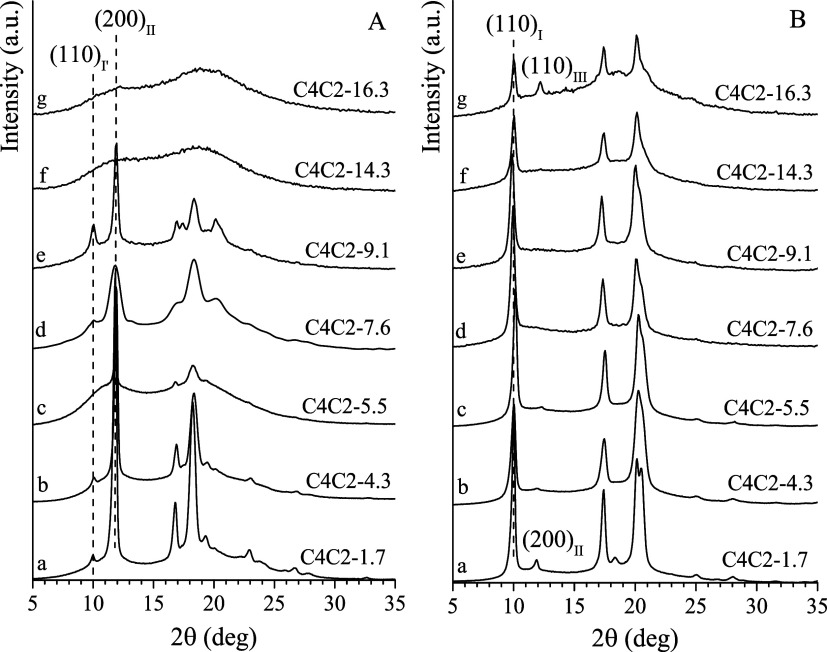
X-ray powder diffraction profiles of unoriented, compression-molded films of C4C2 copolymers recorded immediately after preparation (A, fresh samples) and after prolonged aging at ambient temperature (7 days or more; B, aged samples). The (110)_I_, (200)_II_ and (110)_III_ reflections of form I/I′, form II and form III of iPB are indicated.

For copolymers with C2 contents below 7.6 mol % (samples C4C2–1.7, C4C2–4.3, C4C2–5.5 and C4C2–7.6), crystallization from the melt occurs predominantly in the form II of iPB, as evidenced by the presence of (200)_II_, (220)_II_ and (213)_II_+(311)_II_ reflections of form II,^71^ appearing at 2θ angles of 11.9°, 16.9°, and 18.4°, respectively, in profiles a-d of Figure 1A. A very small amount of form I′ (approximately 4–5%, Table S2) is present in samples C4C2–1.7, C4C2–4.3, and C4C2–7.6, as indicated by the presence of the (110)_I_ reflection of form I^72^ at 2θ = 10° in profiles a, b, and d of Figure 1A. Copolymer with 9.1 mol % ethylene (sample C4C2–9.1) crystallizes into a mixture of form I′ and form II. This is confirmed by the presence of both form II reflections^71^ and (110)_I_, (300)_I_, and (220)_I_+(211)_I_ reflections of form I^72^ (located at 2θ values of 10°, 17°, and 21°, respectively), in profile e of Figure 1A. In this fresh sample, the percentage of form I′ is approximately 40% (Table S2). Finally, copolymers with higher concentrations of C2 (samples C4C2–14.3 and C4C2–16.3) obtained immediately after melt cooling are essentially amorphous (profiles f and g of Figure 1A) with the sample C4C2–16.3 showing very weak crystallinity from crystals of forms I′ and II (profile g of Figure 1A). A small fraction of form III, probably due to the compression pressure applied during molding, appears in copolymer C4C2–16.3, as indicated by the low-intensity peak at 2θ = 14.2° visible in the aged sample profile (profile g in Figure 1B).

X-ray powder diffraction profiles of compression-molded films obtained after prolonged aging (≥ 7 days) at ambient temperature (Figure 1B) demonstrate that all samples are crystallized in form I/I′, as revealed by the presence of characteristic reflections of form I at 2θ = 10°, 17°, and 21°, corresponding to the (110)_I_, (300)_I_, and (220)_I_+(211)_I_ planes, respectively.^72^ These results indicate a total transformation of form II, originally present in the fresh samples shown in Figure 1A, into form I during aging at ambient temperature. The degrees of crystallinity of the fresh samples C4C2–1.7 and C4C2–4.3 are 60% and 47%, respectively, and do not show a notable increase after aging (Table S2). This implies that in these samples, aging leads only to the crystal-to-crystal conversion from form II to form I, without additional crystallization from the amorphous phase. The time of half-transformation of form II into form I (i.e., the aging time at which 50% of the initial form II transforms into form I), indicated as *t*_1/2 (II–I)_, was found to be approximately 9 and 2.5 h for the samples C4C2–1.7 and C4C2–4.3, respectively (Figure S4). In comparison, *t*_1/2(II–I)_ in the iPB homopolymer was found to be approximately 23 h (Figure S4). These results show that incorporating small amounts of ethylene counits (1.7 and 4.3 mol %) into iPB significantly enhances the polymorphic transition from the kinetically favored tetragonal form II to the thermodynamically stable hexagonal form I, in agreement with previous studies.^52,53^ The half-transformation times are slightly lower than those reported in ref 53, probably due to the fact that the procedure used for the crystallization gives a small amount of form I′ in the fresh compression-molded samples (profiles a, b of Figure 1A), which accelerates the transformation into form I.

The C4C2–5.5 sample, containing 5.5 mol % ethylene, exhibits a different crystallization behavior. As previously mentioned, this sample crystallizes directly into form II upon melt cooling, with an initial crystallinity of 18% (profile c in Figure 1A and Table S2). Aging at ambient temperature induces the transformation of form II to form I (profile c in Figure 1B) and leads to a marked increase in crystallinity from 18% to 45% (Table S2). The increase in crystallinity (from 18% to 38–42%) occurs during the early stages of aging (*t*_a_ = 0.5–1 h) and is linked to further crystallization of form II from the amorphous phase (Figure S5A). Then, after about 2 h, the conversion from form II to form I begins (Figure S5A). The time of half-transformation of form II into form I (*t*_1/2 (II–I)_) results equal to 4 h (Figure S5B).

The aging at ambient temperature of samples C4C2–7.6, C4C2–9.1, and C4C2–16.3 (that crystallize immediately after melt cooling in mixtures of form I′ and form II, profiles d, e, and g in Figure 1A) also produces both an increase of crystallinity (Table S2) and a transition of form II into form I. The detailed investigation of the crystallization behavior of these samples, reported in ref 60, revealed that for these copolymers the increase of crystallinity is due to further crystallization of form I′ from the amorphous phase. The time of half-transformation of form II into form I (*t*_1/2 (II–I)_) in the samples C4C2–7.6, C4C2–9.1, and C4C2–16.3, reported in ref 60 in crystallization conditions different from those used in this work, is equal to 2.0, 1.3, and 4.6 h, respectively.

Finally, the sample C4C2–14.3, containing 14.3 mol % ethylene, remains amorphous immediately after melt cooling (profile f in Figure 1A) and undergoes crystallization into form I′ upon storage at ambient temperature (profile f in Figure 1B).

The results obtained for copolymers with a C2 content ≥5.5 mol % confirm that the incorporation of ethylene counits into iPB significantly accelerates the form II-to-form I transition. In fact, the *t*_1/2 (II–I)_ values for samples C4C2–5.5, C4C2–7.6, C4C2–9.1, and C4C2–16.3 are 4, 2, 1.3, and 4.6 h, respectively. In comparison, as previously stated, the time of half-transformation of form II into form I in the iPB homopolymer is approximately 23 h. However, the *t*_1/2 (II–I)_ values observed in these Ziegler–Natta C4C2 copolymers do not show a clear correlation with the ethylene content. This is attributed to the complex molecular architecture of these materials. Specifically, Ziegler–Natta isotactic 1-butene-ethylene copolymers consist of fractions characterized by “segments” containing long regular butene sequences and low ethylene content, and “segments” with shorter regular butene sequences and higher ethylene content.^58,59^ Because of this “blocky-like” structure, the kinetics of the form II-to-form I transition in Ziegler–Natta C4C2 copolymers is governed by the relative abundance of the different fractions having different lengths of the regular butene sequences, rather than by the overall average ethylene content.^60^

The mechanical stress–strain curves of compression-molded films of C4C2 copolymers collected after preparation by cooling from the melt (fresh samples, Figure 1A) and after prolonged aging at ambient temperature (aged samples, Figure 1B) are reported in Figure 2A,B, respectively. In the case of the low-crystalline sample C4C2–5.5 (profile c of Figure 1A) and amorphous sample C4C2–14.3 (profile f of Figure 1A), the stress–strain curves reported in Figure 2A were acquired on films aged at ambient temperature for 1 h (Figure S6) to allow complete crystallization of the sample C4C2–5.5 in form II with a degree of crystallinity equal to 42% (Figure S6A and Table S2), and crystallization of the sample C4C2–14.3 in form I′ with a degree of crystallinity of 15% (Figure S6B and Table S2). Mechanical parameters of fresh and aged samples, determined from stress–strain curves in Figure 2A,B, respectively, are reported in Table S2 and plotted as a function of C2 concentration in Figure S7. Mechanical parameters for a sample of iPB homopolymer synthesized with the same catalytic system used for the C4C2 copolymers^21^ are added for comparison in Figure S7. The stress–strain behavior depicted in Figure 2 reveals that both fresh and aged samples display similar mechanical characteristics, indicative of materials that are flexible and ductile. Noticeable differences emerge only at low strain values, specifically in Young′s modulus (Figure S7A) and yield stress (Figure S7B), with aged compression-molded films showing values higher than those of their freshly prepared counterparts. This increase is due to the transition of form II into form I during aging, as form I crystals are known to exhibit higher stiffness and yield strength.^22,57^

**Figure 2:**
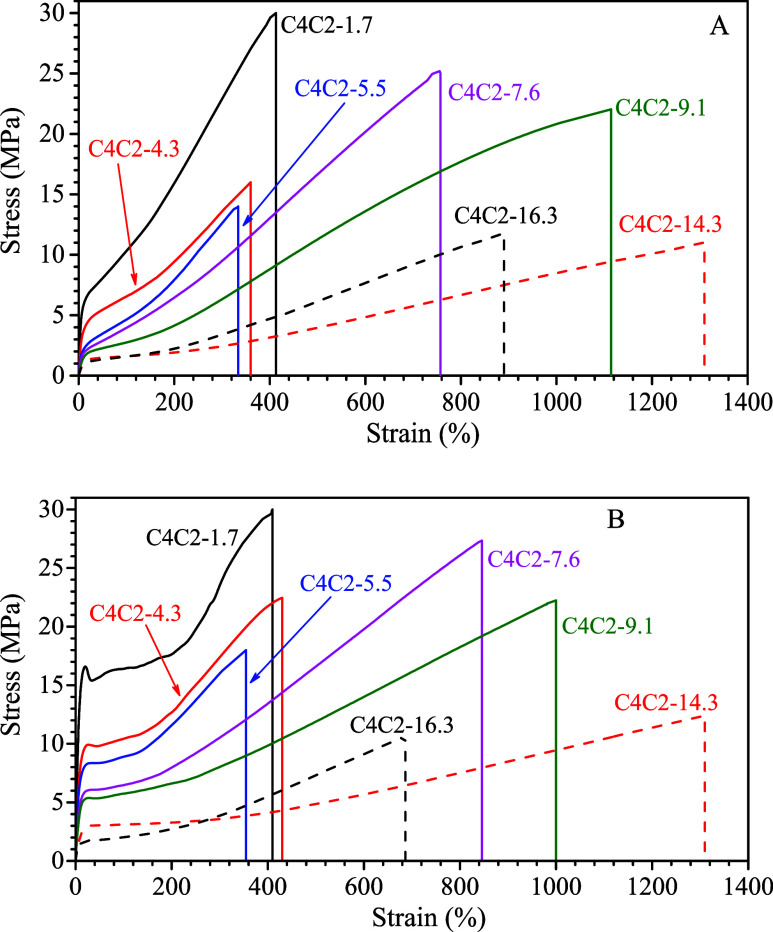
Mechanical stress–strain curves of compression-molded films of C4C2 copolymers immediately after preparation by cooling from the melt (A) and after prolonged aging at ambient temperature (B). The curves of samples C4C2–5.5 and C4C2–14.3 reported in A were acquired on films aged at ambient temperature for 1h (Figure S6).

For copolymers containing ethylene up to 9.1 mol %, the distinction in tensile performance between fresh and aged films is more pronounced. These materials crystallize primarily into form II or into a mix of form II and form I′, with form II being dominant (Figure 1A and Table S2). Upon aging, a significant conversion of form II into form I occurs. Consequently, freshly molded samples deform uniformly without neck formation (Figure 2A), while aged samples exhibit clear necking during stretching (Figure 2B). In contrast, copolymers with higher ethylene contents, 14.3 and 16.3 mol % (samples C4C2–14.3 and C4C2–16.3), exhibit comparable stress–strain curves before and after aging. These samples undergo rubber-like deformation without neck formation in both conditions (Figure 2A,B), and only a slight increase in Young′s modulus and yield stress is observed in aged specimens (Figure S7A,B, and Table S2). This behavior is explained by their initial low crystallinity (Table S2), with small amounts of form II and/or form I′, and by the fact that aging leads mainly to increased crystallinity of form I′, without a significant transformation from form II to form I. At higher strains, both fresh and aged samples demonstrate comparable mechanical responses regardless of the ethylene content or initial crystalline structure. This is supported by the similar values observed for stress and strain at break (σ_b_ and ε_b_, Figure S7D,E). Overall, the incorporation of ethylene into iPB chains influences the tensile behavior by enhancing flexibility and drawability while reducing stiffness. Indeed, as ethylene content increases, a reduction in Young’s modulus (Figure S7A), yield stress, and break stress (Figure S7B,D) is observed, along with an increase in strain at yield and at break (Figure S7C,E), in both freshly prepared and aged samples.

### Crystallization Behavior during Tensile Deformation

Unoriented compression-molded films for X-ray diffraction measurements with synchrotron radiation during tensile deformation were prepared by melting samples at temperatures 30–40 °C higher than their melting temperature for 2–3 min and cooling to ambient temperature by air quenching. One-dimensional diffraction profiles of the initial unoriented films of C4C2 copolymers are reported in Figure S8. Degrees of crystallinity (*x*_c_) and percentages of crystals of form II (*f*_II_) and form I′ (*f*_I′_) in films of C4C2 copolymers immediately after cooling from the melt (unoriented sample, Figure S8) are reported in Table 2. The profile of the sample C2C4–16.3 (Figure S8G) was acquired after aging the film at ambient temperature for ≈ 2 h. According to the data reported in Figure 1A, samples with C2 content ≤7.6 mol % (samples C4C2–1.7, C4C2–4.3, C4C2–5.5 and C4C2–7.6, Figure S8A–D and Table 2) are crystallized in form II with only a very low amount of form I′ in the case of the samples C4C2–4.3 (*f*_I′_ = 6%, Figure S8B and Table 2) and C4C2–7.6 (*f*_I′_ = 3%, Figure S8 D and Table 2). Sample C4C2–9.1 with a C2 content of 9.1 mol % is crystallized in a mixture of form II and form I′ with a percentage of crystals of form I′ with respect to the crystals of form II of 49% (Figure S8E and Table 2). Finally, sample C4C2–14.3 is basically amorphous (Figure S8F), and sample C2C4–16.3 aged at ambient temperature for ≈ 2 h is crystallized in a mixture of form I′ and form II with a percentage of crystals of form I′ with respect to the crystals of form II equal to 65% (Figure S8G and Table 2). As already stated, these copolymer samples show a “blocky-like” structure and consist of fractions characterized by “segments” containing long regular butene sequences and low ethylene content, and “segments” with shorter regular butene sequences and higher ethylene content. Therefore, the reported results indicate that the crystallization behavior of these systems is different from that of a truly random copolymer, but in any case, it is also different from that of simple blends of butene-ethylene copolymers with high and low incorporation rates.

**Table 2:** Percentages of Crystals of Form II (*f*_II_) and Form I′ (*f*_I′_) in Films of C4C2 Copolymers Immediately after Cooling from the Melt (Unoriented Sample, Figure S8), and Percentages of Crystals of Total Form I (*f*_(I+I')_, eq S1), Form I Originated from Transition of Form II (*f*_I(II)_) (eq S4), Form I′ Obtained from Crystallization of the Amorphous Phase (*f*_I′(am)_) (eq S5) and Residual Form II (*f*_II_) in Stretched films^a^

		unoriented films			stretched films^c^						
sample	C2 (mol %)	*x*_c_ (%)	*f*_II_ (%)	*f*_I'_ (%)	*x*_c_ (%)	*f*_(I+I′)_ (%)	*f*_I(II)_ (%)	*f*_I′(am)_ (%)	*f*_II_ (%)	ε_c_ (%)	ε_0.5_ (%)
C4C2–1.7	1.7	59	100	0	59	97	97	0	3	8	58
C4C2–4.3	4.3	48	94	6	55	96	78	18	4	12	89
C4C2–5.5	5.5	23	100	0	41	68	24	44	32	≈ 80	-
C4C2–7.6	7.6	36	97	3	40	95	82	13	5	16	94
C4C2–9.1	9.1	42	51	49	43	95	45	50	5	80	239
C4C2–16.3^b^	16.3	18	35	65	27	93	16	77	7	90	≈344

a The values of the critical strain at which the form II–form I transition begins (ε_c_), the strain at which 50% of the initial form II transforms into form I (ε_0.5_), and the degrees of crystallinity (*x*_c_) of unoriented and stretched films are also indicated.

b Data acquired after aging the film at room temperature for ≈ 2 h.

c Data obtained at the maximum strains (ε) explored during the in situ WAXD experiments, i.e., ε = 261% for samples C4C2–1.7 and C4C2–4.3, 500% for the sample C4C2–5.5, 265% for the sample C4C2–7.6, 380% for the sample C4C2–9.1, and 344% for the sample C4C2–16.3.

Unoriented films (Figure S8) were stretched by uniaxial deformation up to a strain value comprised in the range 270–500% (depending on the copolymer), and two-dimensional (2D) wide-angle X-ray diffraction (WAXD) patterns were collected during deformation. Data of the C4C2–14.3 copolymer are not shown since the amorphous immediately prepared C4C2–14.3 sample (Figure S8F) resulted in sticky specimen, not allowing for recording the stress–strain curve. It is worth noting that stress–strain curves and, consequently, in situ WAXD patterns were acquired in a maximum time comprised between 122 and 225 s (depending on the sample). The degrees of crystallinity (*x*_c_) and the percentages of the different crystalline phases in stretched films, evaluated by using the procedure described in Supporting Information, are reported in Table 2.

Selected 2D WAXD patterns and corresponding 1D diffraction profiles recorded during stretching of the samples C4C2–1.7, C4C2–4.3, and C4C2–5.5 (Figure S8 A–C) are reported in Figures 3, 4, and 5, respectively. In panels A of Figures 3, 4, and 5, the tensile stretching axis lies along the 0–180° (horizontal) direction of the 2D WAXS patterns. Accordingly, the azimuthal angles of 0° and 90° correspond to the meridional and equatorial directions, respectively.

**Figure 3:**
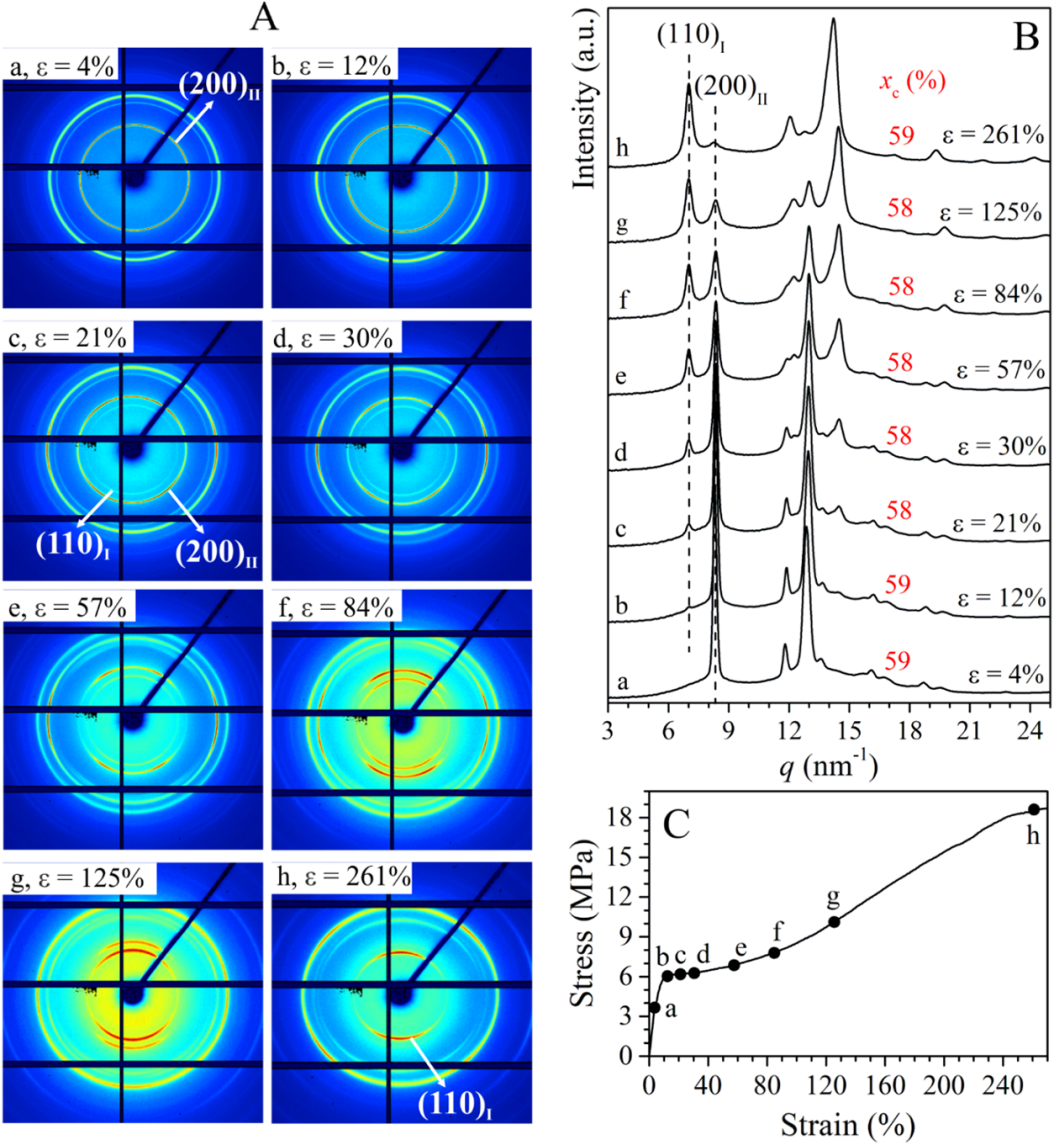
Selected 2D WAXD patterns (A) and the corresponding 1D diffraction profile integrated over the azimuthal coordinate χ (B), recorded during the stretching of sample C4C2–1.7 with 1.7 mol % of ethylene at the indicated values of strain ε, corresponding to points a-h of the stress–strain curve (C). The tensile stretching axis lies along the 0–180° (horizontal) direction of the 2D WAXS patterns. In (B), the degrees of crystallinity *x*_c_ are indicated.

**Figure 4:**
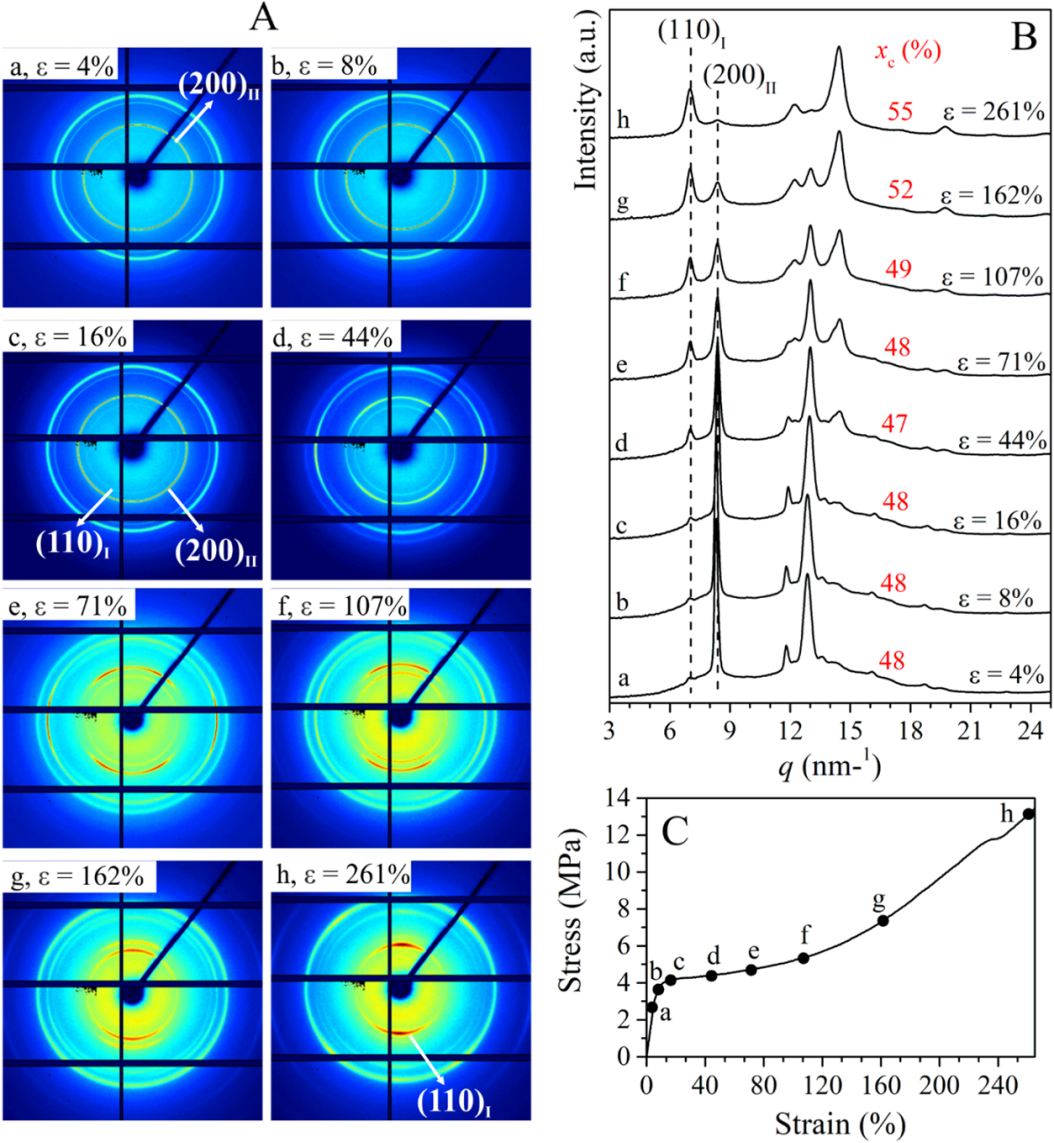
Selected 2D WAXD patterns (A) and corresponding 1D diffraction profile integrated over the azimuthal coordinate χ (B), recorded during the stretching of sample C4C2–4.3 with 4.3 mol % of ethylene at the indicated values of strain ε, corresponding to points a–h of the stress–strain curve (C). The tensile stretching axis lies along the 0–180° (horizontal) direction of the 2D WAXS patterns. In (B), the degrees of crystallinity *x*_c_ are indicated.

**Figure 5:**
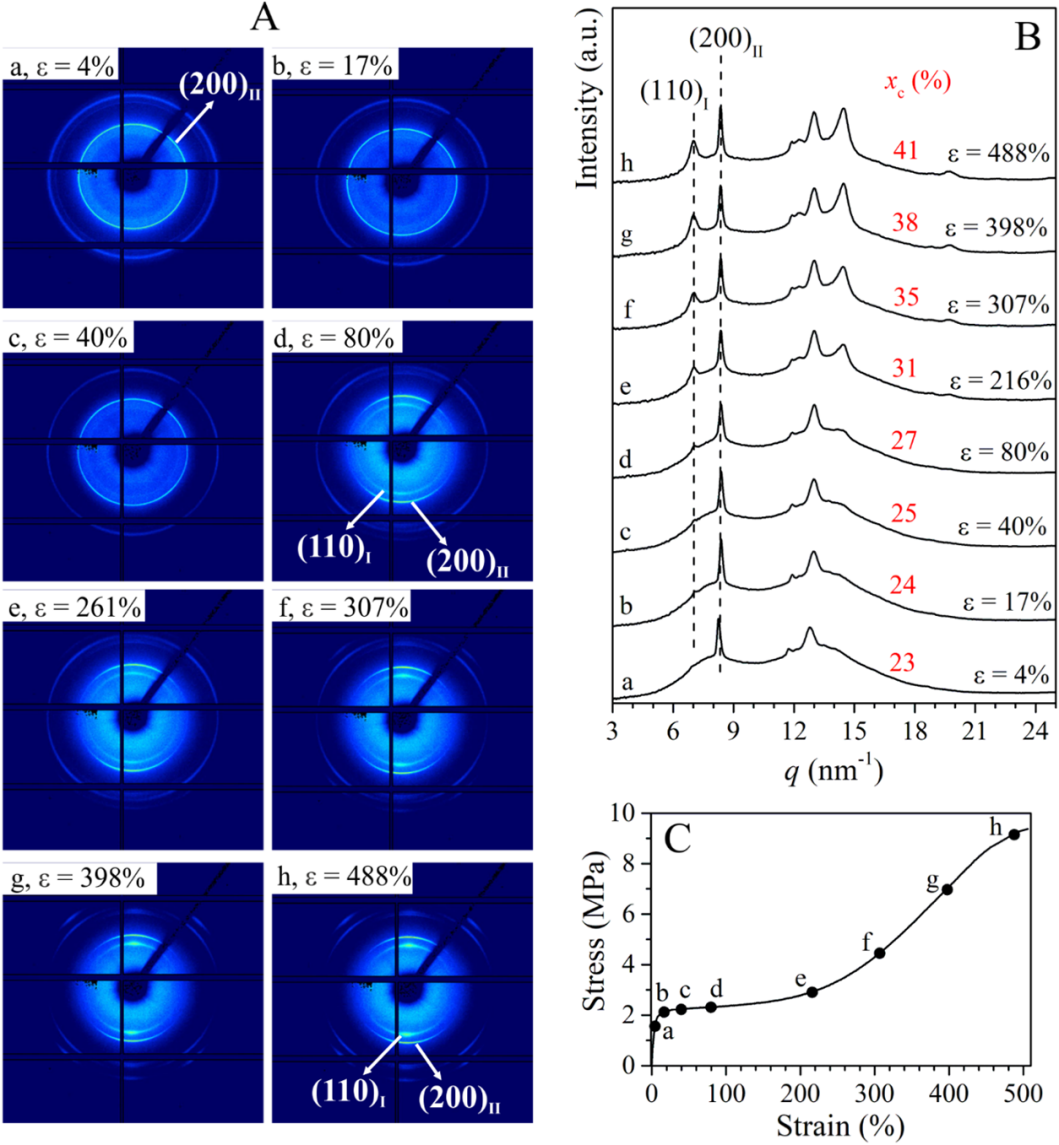
Selected 2D WAXD patterns (A), and corresponding 1D diffraction profile integrated over the coordinate azimuthal χ (B), recorded during the stretching of sample C4C2–5.5 with 5.5 mol % of ethylene at the indicated values of strain ε, corresponding to points a–h of the stress–strain curve (C). The tensile stretching axis lies along the 0–180° (horizontal) direction of the 2D WAXS patterns. In (B), the degrees of crystallinity *x*_c_ are indicated.

The WAXD pattern of the sample C4C2–1.7 acquired at very low deformation (ε = 4%, Figure 3a) presents only (200)_II_, (220)_II_, and (213)_II_ + (311)_II_ reflections of form II at *q* ≈ 8.4, 12.0, and 12.9 nm^–1^, respectively. As stretching proceeds (Figure 3 b–h), new diffraction peaks at *q* ≈ 7.0, 12.3, and 14.4 nm^–1^ corresponding to (110)_I_, (300)_I_, and (220)_I_ + (211)_I_ reflections of form I appear. The degree of crystallinity *x*_c_ is constant during stretching (Figure 3B), indicating that form I appearing during stretching originates from the transformation of form II present in the initial unoriented sample. Therefore, as stretching proceeds (Figure 3 b–h), form II present in the initial sample gradually transforms into the thermodynamically stable form I. The absence of form I in the data acquired before yielding (Figure 3a) indicates that form II–form I transition starts after a critical point corresponding to the yielding point in stress–strain mechanical curve, as already reported in the case of iPB homopolymer.^35–37,41,42,63,64^ The degrees of crystallinity *x*_c_, and values of the fractions of form II (*f*_II_^′^) and of form I obtained from transformation of form II (*f*_I_^**′**^) with respect to the total mass of the sample, calculated by using eqs S2 and S3, are reported in Figure 6A as a function of strain. The percentage of crystals of form I obtained from the transformation of form II with respect to the crystals of form II, calculated by eq S1, is reported in Figure 6A′ as a function of strain, superposed to the mechanical stress–strain curve of the sample. The data of Figure 6A′ indicate that *f*_I_ starts increasing at deformation values near the yielding point in the mechanical curve, confirming that no form II–form I transition occurs before the yielding point (stage 1 in Figure 6A′). Then, II–I phase transition evolves quickly up to a strain of ≈ 125%, reaching a percentage of form I equal to ≈ 80% at this deformation (stage 2 in Figure 6A′), and more slowly at higher values of strain (stage 3 in Figure 6A′). The transition is essentially complete at a strain of around 260% (where *f*_I_ = 97%, Table 2). Clearly, stretching strongly accelerates II–I phase transition, since for the unoriented sample C4C2–1.7 more than 5 days are required to fully complete form II–form I transition, and the aging time at which 50% of the initial form II transforms into form I (*t*_1/2 (II–I)_) is equal to ≈ 9 h (curve b in Figure S4). In parallel with crystal–crystal transition, the orientation of crystals of both the original form II and the transformed form I occurs during stretching. In particular, the WAXD data (Figure 3A) indicate that crystals of both form II and form I orient during early stretching with the polymer chain axis tilted relative to the stretching direction.

**Figure 6:**
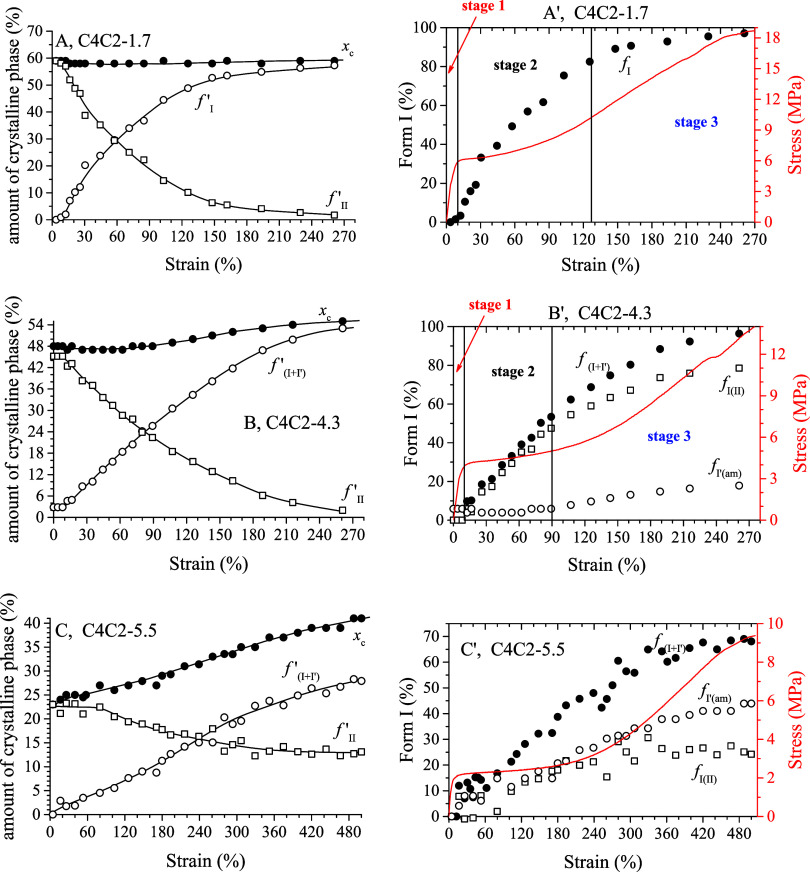
Values of crystallinity (*x*_c_), fractions of total form I (*f*_(I+I′)_^′^, eq S2) and form II (*f*_II_^′^, eq S3) with respect to the total mass of the samples (A–C) and fractions of the crystallinity *x*_c_ given by crystals of total form I (*f*_(I+I′)_, eq S1), form I originated from transition of form II (*f*_I(II)_) (eq S4) and form I′ obtained from crystallization of the amorphous phase (*f*_I′(am)_) (eq S5) (A′–C′) for the samples C4C2–1.7 (A, A′), C4C2–4.3 (B, B′) and C4C2–5.5 (C, C′) as a function of strain. For the sample C4C2–1.7 (A, A′), *f*_(I+I′)_^′^= *f*_I_^′^ and *f*_(I+I')_ = *f*_I_. The stress–strain curves of the samples are also reported in A′–C′.

WAXD data of copolymer C4C2–4.3 (Figure 4) indicate that at very low deformation, the sample is still unoriented (ε = 4%, Figure 4a) and crystallized in form II with a very low amount of form I′ (equal to 6%), as the starting sample before stretching (Figure S8B). As stretching proceeds, diffraction peaks of form I gradually appear (Figure 4b–h) and a slight increase in the degree of crystallinity from ≈48 to ≈55% is observed (Figure 4B), indicating that crystallization from the amorphous phase occurs during stretching.

For the sample C4C2–5.5 (Figure 5), which is crystallized as soon after cooling from the melt in form II (Figure S8C and Table 2), a significant increment of crystallinity from 23 to 41% is observed during stretching (Figure 5B and Table 2), in parallel to the appearance of form I/I′ reflections (Figure 5A,B). The values of degree of crystallinity *x*_c_, and the values of the fractions of total form I (form I + form I') and of form II with respect to the total mass of the samples (*f*_(I+I')_^′^ and *f*_II_^′^, respectively), calculated by eqs S2 and S3, are plotted as a function of strain in Figure 6B,C for the samples C4C2–4.3 and C4C2–5.5, respectively. The percentage of crystals of total form I (form I + form I') (*f*_(I+I')_) with respect to the crystals of form II, determined by eq S1, and the fractions of the crystallinity given by the crystals of form I obtained from transformation of form II (*f*_I(II)_) and by crystals of form I′ obtained from crystallization of the amorphous phase (*f*_I′(am)_), calculated as described in the Supporting Information (eqs S4 and S5), are plotted as a function of strain ε in Figure 6B′,C′ for the samples C4C2–4.3 and C4C2–5.5, respectively, superimposed to the mechanical stress–strain curves of the samples. In the case of the sample C4C2–4.3, data of Figure 6B indicate that the degree of crystallinity is constant at *x*_c_ ≈ 48% up to a deformation ε ≈ 90% and then starts increasing. Also, data in Figure 6B′ indicate that no form II–form I transition occurs before yielding (stage 1 in Figure 6B′), as in the case of the sample C4C2–1.7 (Figures 3 and 6 A,A′). After yielding point, only transition of form II into form I occurs up to a strain equal to ≈ 90% (stage 2 in Figure 6B′), and then, at higher strains, form I′ starts crystallizing from the amorphous phase, in parallel with the occurrence of form II–form I transition (stage 3 in Figure 6B′). At 260% strain, sample C4C2–4.3 is fully crystallized in form I/form I′ (*f*_(I′+I)_ = 97%, Figure 6B′ and Table 2) with crystals mainly originating from form II–form I transition (*f*_I(II)_ = 79%, Figure 6B′ and Table 2). Also for this sample, stretching strongly accelerates form II–form I transition, since in the case of the unoriented sample C4C2–4.3 the half-transformation time *t*_1/2(II–I)_ results equal to 2.5 h (curve c in Figure S4).

The sample C4C2–5.5 (Figures 5 and 6 C,C′) exhibits a different behavior upon stretching. For this sample, in fact, during stretching mainly crystallization of form I′ from the amorphous phase occurs and only a low amount of form II present in the initial sample transforms into form I (Figure 6C,C′). The degree of crystallinity starts increasing already at low deformation (Figure 6C), indicating that crystallization of form I′ from the amorphous phase during stretching already occurs at low deformation values (opened circles in Figure 6C′). At strains higher than ≈200%, *f*_I′(am)_ is higher than *f*_I(II)_ (Figure 6C′), indicating that the amount of crystals of form I′ obtained from crystallization of the amorphous phase during stretching is higher than that of crystals of form I that originate from transition of form II. At the highest explored strain (ε = 500%), a significant amount of form II is still present, and only 24% of form II initially present in the sample transforms into form I (Figure 6C′ and Table 2).

We performed the same quantitative analysis for the stretching of the C4C2 copolymers with C2 content higher than 5.5 mol % (Figures S9–S11 and 7). As previously discussed, the initial unoriented C4C2–7.6 sample is predominantly crystallized in form II (Figure S8D and Table 2). Upon stretching this sample up to a strain ε = 265% (Figure S9), the overall degree of crystallinity remains nearly constant (Figures S9B, 7A, and Table 2), the form II–form I transition predominantly occurs, with only a negligible amount of form I′ crystallizing from the amorphous phase (Figure 7A′). The form II–form I transition begins after yielding and is essentially complete at ε = 265%, with 85% of the crystalline form I originating from the transformation of form II (*f*_I(II)_= 85%, Figure 7A′ and Table 2). The unoriented C4C2–9.1 sample is initially crystallized as a mixture of form II and form I′, with a percentage of crystals of form I′ equal to 49% (Figure S8E and Table 2). During stretching (Figures S10 and 7B,B′), only form II–form I transition occurs, and the form II present in the initial sample (*f*_II_ = 51%, Table 2) is almost completely transformed into form I at strain ε = 380% (*f*_I(II)_ = 45%, Figure 7B′ and Table 2). Stretching accelerates the form II to form I transition in both C4C2–7.6 and C4C2–9.1 samples, since the time of half-transformation of form II into form I (*t*_1/2 (II–I)_) under quiescent conditions was estimated to be approximately 2.0 h and 1.3 h for samples C4C2–7.6 and C4C2–9.1, respectively.^60^ It is worth noting that due to limitations of the experimental setup, the maximum strains ε reached during the in situ experiments on samples C4C2–7.6 and C4C2–9.1—approximately 270% and 380%, respectively (Figure S9C and S10C)—are significantly lower than the breaking strain of the samples (Table S2 and Figure 2A). Therefore, for these two samples, crystallization of form I′ at higher strain values during stretching cannot be ruled out. Finally, during stretching up to a strain ε = 345% of the low-crystalline C4C2–16.3 sample, containing a significant amount of form I′ in the initial unoriented state (Figure S8G and Table 2), an increase in crystallinity from 18 to 27% is observed (Figures S11B, 7C and Table 2). For this sample, both further crystallization of form I′ from the amorphous phase and transformation of form II into form I occur upon stretching (Figure 7C′ and Table 2). In particular, at the maximum explored strain (ε = 345%), approximately 50% of form II initially present in the sample transforms into form I (Figure 7C′ and Table 2).

**Figure 7:**
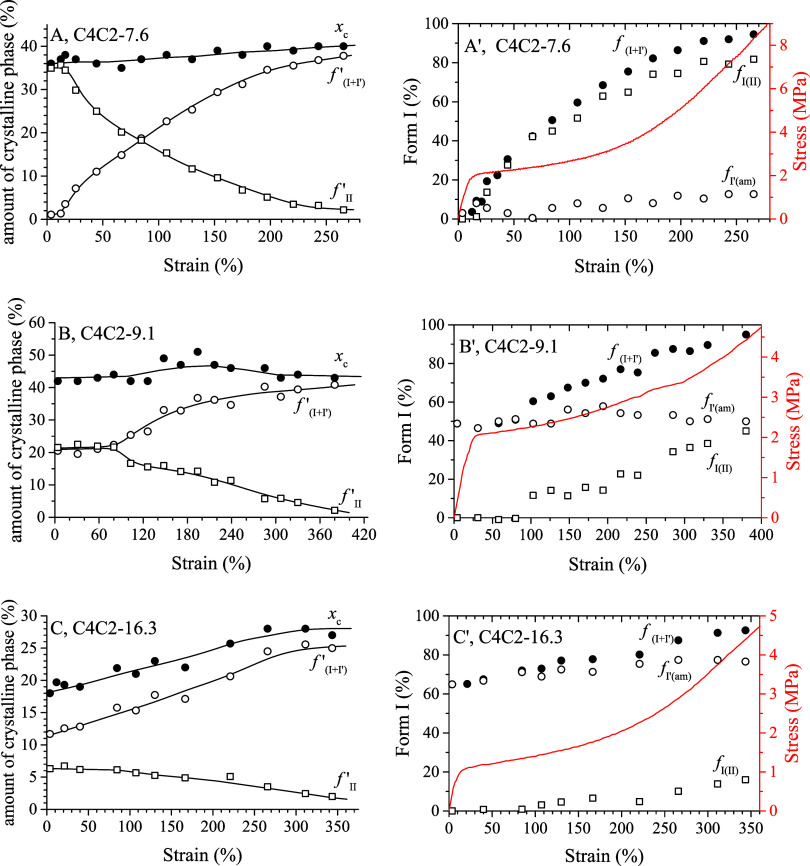
Values of crystallinity (*x*_c_), fractions of total form I (*f*_(I+I′)_^′^, eq S2) and form II (*f*_II_^′^, eq S3) with respect to the total mass of the samples (A–C) and fractions of the crystallinity *x*_c_ given by crystals of total form I (*f*_(I+I')_, eq S1), form I originated from transition of form II (*f*_I(II)_, eq S4) and form I′ obtained from crystallization of the amorphous phase (*f*_I′(am)_) (eq S5) (A′–C′) for the samples C4C2–7.6 (A, A′), C4C2–9.1 (B, B′), and C4C2–16.3 (C, C′) as a function of strain. The stress–strain curves of the samples are also reported in A′- C′.

A comparison of crystallinity and percentages of the different crystalline phases measured during the stretching of C4C2 copolymers is shown in Figure S12. The data in Figure S12B show that all samples, except the copolymer containing 5.5 mol % ethylene (sample C4C2–5.5, Figure S12B), are basically crystallized in form I/I′ at high strains (ε ≥ 261%). In fact, the total amount of form I (*f*_(I+I′)_) in the stretched samples reaches or exceeds 93% (Figure S12B and Table 2). During stretching at room temperature, a direct transformation from metastable form II to stable form I takes place. The applied tensile stress aligns and extends the helices, facilitating helical pitch adjustment, contraction, and interchain slippage required for the form II-to-form I transition.^35^ In addition, tensile deformation generates further nucleation sites for form I by enhancing local thermal stress and promoting stress transfer from the “activated” amorphous chains to the lamellae.^66^

From the data of Figure S12C, the values of the critical strain at which the form II–form I transition begins (ε_c_) and the strain at which 50% of the initial form II transforms into form I (ε_0.5_) were determined for each sample. Specifically, the value of ε_c_ corresponds to the minimum strain at which form I originating from the transition of form II (*f*_I(II)_) is detected or, equivalently, to the strain at which the amount of form II present in the initial unstretched sample begins to decrease (i.e., *f*_II_^′^ in Figures 6A,B and 7A–C). The values of ε_c_ and ε_0.5_ are listed in Table 2 and plotted as a function of the ethylene content in Figure 8. The value of ε_c_ obtained for the iPB homopolymer—synthesized under the same conditions as the C4C2 copolymers and determined from the data reported in Figures S13 and S14—is also included in Figure 8 for comparison. The results for the copolymer C4C2–5.5 are not included since, as previously discussed (Figures 5 and 6C,C′)—for this sample, mainly crystallization of form I′ from the amorphous phase occurs during stretching, and only 24% of form II present in the initial sample transforms into form I (Table 2). The data in Figure 8 and Table 2 show that both ε_c_ and ε_0.5_ increase with increasing C2 content. A significant jump is observed between samples with C2 content ≤7.6 mol % (initially basically crystallized in form II, Figure S8A–D) and those with C2 content >7.6 mol % (initially crystallized as a mixture of form II and form I′, Figure S8 E,G). Specifically, ε_c_ increases from 16% for sample C4C2–7.6 to 80% for sample C4C2–9.1 (Figure 8 and Table 2), and ε_0.5_ increases from 94% for sample C4C2–7.6 to 239% for sample C4C2–9.1 (Figure 8 and Table 2). Additionally, the data of Figure 8 indicate that ε_c_ is close to the strain at yielding in samples with C2 content ≤7.6 mol % (copolymers C4C2–1.7, C4C2–4.3, and C4C2–7.6; see Figures 3, 4, and S9) and much higher than the strain at yielding in samples with higher ethylene content (copolymers C4C2–9.1 and C4C2–16.3; see Figures S10 and S11).

**Figure 8:**
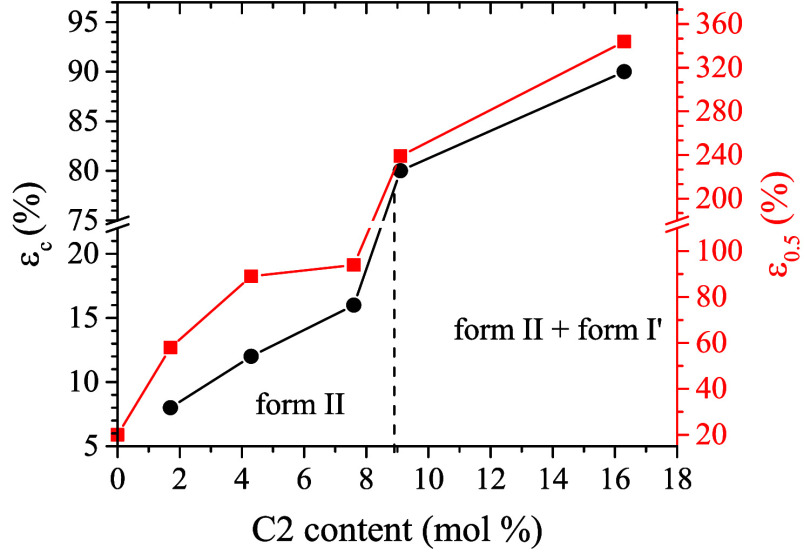
Values of the critical strain at which the form II–form I transition begins (ε_c_, black circles) and strain at which 50% of the initial form II transforms into form I (ε_0.5_, red squares) as a function of ethylene (C2) content. The value of ε_c_ for the iPB homopolymer was evaluated from data reported in Figures S13 and S14.

The increase in the critical strain at which the form II-to-form I transition begins (ε_c_) observed with an increasing ethylene content could be attributed to a decrease in the efficiency of stress transfer from the tie chains to the crystalline lamellae as the ethylene content increases. The “activation” of tie chains plays a key role in triggering the form II-to-form I transition. Under tensile deformation, tie chains transmit mechanical force to the crystalline lamellae, creating local conditions that promote nucleation of form I.^66^ The incorporation of ethylene units into the polybutene backbone decreases the crystallinity, leading to a lower lamellar density and the presence of thinner and more disordered crystalline lamellae. Consequently, larger amorphous regions, and therefore more tie chains, are present between lamellae. Although more numerous, tie chains in copolymers are less effective at transferring stress to lamellae due to (i) the more disordered structure, with less well-developed lamellae to connect; (ii) tie chains being shorter or more entangled; and (iii) a higher amorphous content, which dilutes stress transmission. Since tie chains become less efficient at transmitting mechanical deformation to the lamellae as the ethylene content increases, a higher strain is required to align the chains, disrupt or reorganize the lamellae, and trigger the form II–form I transition. The nonmonotonic behavior—characterized by a jump in ε_c_ between samples with C2 content ≤7.6 mol % and those with C2 content >7.6 mol %—may be attributed to the different crystals present in the initial polymorphic composition of the samples before stretching. Specifically, the samples with C2 content ≤7.6 mol % are initially basically crystallized in form II (Figure S8A,B,D), whereas copolymers with C2 content >7.6 mol % are initially crystallized as a mixture of form II and form I′, with a significant presence of form I′ crystals (Figure S8E,G). The presence of pre-existing form I′ crystals in samples with C2 content >7.6 mol % may constrain the deformation of neighboring form II domains, either mechanically—by restricting chain mobility—or topologically—by reducing the cooperative nature of the transition. As a result, the strain required to activate the transition increases sharply for these samples.

As previously stated, the analyzed copolymers consist of fractions characterized by “segments” containing long regular butene sequences with low ethylene content and “segments” with shorter regular butene sequences and higher ethylene content. This not fully random comonomer distribution leads to the presence of butene sequences longer than expected (based on the overall concentration of ethylene), which allows crystallization of form II in the unstretched state, even at relatively high ethylene content. In contrast, metallocene C4C2 copolymers characterized by a perfectly random distribution of ethylene show a completely different crystallization behavior.^57^ In these samples, in fact, the perfectly random comonomer distribution makes the regular butene sequences very short even at low ethylene contents, resulting in experimental evidence that metallocene copolymers crystallize in form I′ from the melt even at low ethylene concentrations, and are unable to crystallize at slightly higher ethylene contents,^57^ at which ZN copolymers still crystallize in form II. This completely different crystallization behavior between ZN and metallocene copolymers prevents a direct comparison of the stress-induced phase transformations occurring in ZN and metallocene samples at the same ethylene concentrations to extract the effect of the ethylene distribution.

The 2D WAXD patterns of the copolymers recorded at strain values equal to the onset of phase transformation (ε_c_) are reported in Figure S15. The patterns of all samples (Figure S15A) exhibit homogeneous azimuthal intensity distributions of the (200)_II_ reflection of form II, indicating that form II crystals remain unoriented at ε_c_. The orientation of form II crystals therefore begins only after the onset of the form II-to-form I transition. In the case of samples C4C2–9.1 and C4C2–16.3 (data d and e in Figure S15, respectively), the form I′ crystallized from the amorphous phase is also still unoriented at ε_c_ (patterns d and e in Figure S15A). As stretching proceeds, in parallel with form II-to-form I transition and with crystallization of form I′ from the amorphous phase in the case of samples C4C2–4.3 and C4C2–16.3, the orientation of crystals occurs. Specifically, for samples C4C2–1.7, C4C2–4.3, and C4C2–7.6, the orientation of the original form II, of the transformed form I, and – only in the case of sample C4C2–4.3 – of form I′ crystallized from the amorphous phase, is observed. This is demonstrated by the nonhomogeneous azimuthal intensity distributions of the (200)_II_ reflection and (110)_I_ reflection of form I/I′ in 2D WAXS patterns of these samples acquired at higher strains (panels A in Figures 3, 4, and S9). In contrast, for samples with higher C2 content (samples C4C2–9.1 and C4C2–16.3), the form II crystals remain essentially isotropic throughout the stretching process in the explored strain range and only orientation of the form I/I′ crystals is detected (Figures S10 and S11). The orientation degree of form II as a function of strain for samples C4C2–1.7, C4C2–4.3, and C4C2–7.6 was evaluated by calculating the Hermans’ orientation function from the azimuthal intensity distributions of the (200)_II_ reflection of form II (see Supporting Information). The results (Figure S16) indicate that, concurrently with the form II-to-form I transition, residual form II crystallites, initially isotropic, gradually approach an equatorial orientation as the strain increases. As previously mentioned, the orientation of form II crystals in each sample begins only after the onset of the form II-to-form I transition. Subsequently, the evolution of orientation occurs in a similar manner for all samples (Figure S16D), suggesting that the degree of orientation of form II crystals is mainly governed by the strain level and not by the progression of the phase transformation process.

The determined values of the critical strain at which the form II- form I transition begins (ε_c_, black circles in Figure 8), together with the data in Figure S12B, enabled the construction of the phase diagram shown in Figure 9 that define the regions of stability of the different polymorphic forms as a function of ethylene content and deformation at the used strain rate. In particular, the boundary line between the form II region (or (form II + form I′) region in the case of the samples with higher C2 content) and the (form II + form I) region was determined using the values of ε_c_ (black circles in Figures 8 and 9). The boundary line between the (form II + form I) region and form I region was instead identified based on the values of strain at which the amount of total form I in the samples (*f*_(I+I')_), Figure S12B) reaches or exceeds 93% (open squares in Figure 9). This threshold was chosen based on the values of *f*_(I+I')_ obtained in stretched films, which lie within the range of 93–97%, depending on the copolymer (see Table 2). Therefore, to include all samples in the phase diagram, the threshold for defining the form I region in Figure 9 was set to a minimum of 93% form I + form I′ content. It is worth noting that the strains required for complete conversion (*f*_(I+I′)_ = 100%), obtained by extrapolating the fitted curves of Figure S12B (see Figure S17), are ≈360, 330, 420, 670, and 650% for samples C4C2–1.7, C4C2–4.3, C4C2–7.6, C4C2–9.1, and C4C2–16.3, respectively. Thus, the strain required for complete conversion roughly increases with increasing C2 content in the copolymer, showing a significant jump (Δε ≈ 240%) between samples with C2 content ≤7.6 mol % and those with C2 content >7.6 mol %. This trend is similar to that observed for the values of ε_c_, ε_0.5_ (Figure 8), and for the strain at which the amount of total form I reaches or exceeds 93% (open squares in Figure 9). In the phase diagram of Figure 9, the extrapolated strain values at which the amount of form I would reach 100% are also included (open circles in Figure 9). The extrapolated boundary line between the form II + form I region and the form I region lies at strain values only slightly higher than those of the available experimental data.

**Figure 9:**
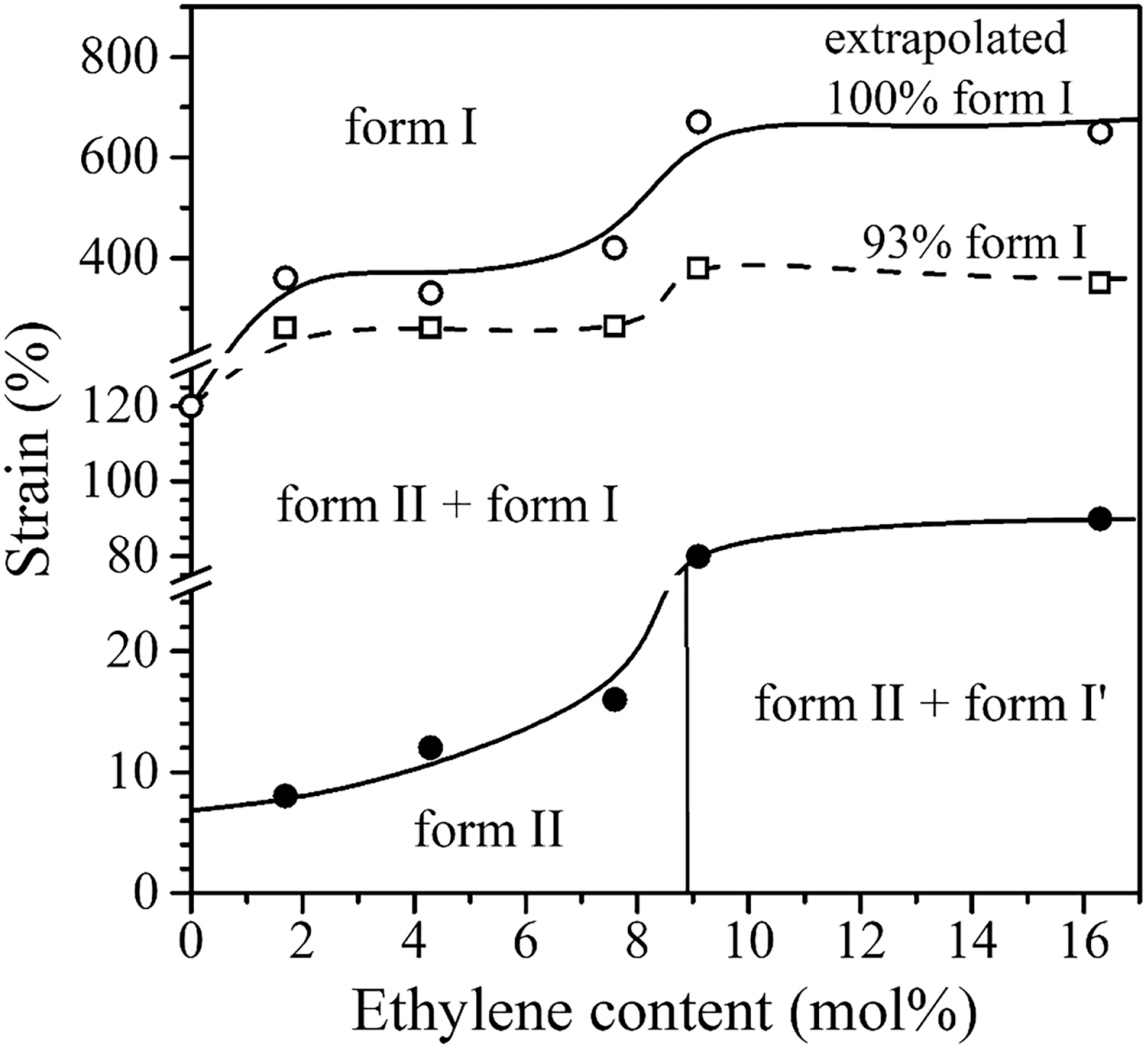
Phase diagram of C4C2 copolymers as a function of strain and ethylene (C2) content. The experimental strain values at which the total amount of form I in the samples (*f*_(I+I')_) reaches or exceeds 93% are indicated with open squares (93% form I). The extrapolated strain values at which the amount of form I would reach 100% are indicated with open circles.

The phase diagram in Figure 9 indicates that, as previously discussed, higher values of strain are required to trigger the form II- form I transition with increasing C2 content. Additionally, samples that are basically crystallized in form I/I′ (*f*_(I+I')_ ≥ 93%) can be obtained at strain values above ≈ 260% for copolymers with lower ethylene content. In contrast, higher strain values (≥350%) are needed to achieve samples basically crystallized in form I/I′ in the case of copolymers with higher ethylene content.

## Conclusions

Phase transitions and crystallization behavior during stretching of random C4C2 copolymers, synthesized using a Ziegler–Natta catalyst and characterized by an ethylene (C2) content between 1.7 and 16.3 mol %, were investigated using a combination of tensile testing and *in situ* wide-angle X-ray diffraction (WAXD) with synchrotron radiation. The samples exhibited a stress–strain behavior characteristic of flexible and ductile materials and showed enhanced flexibility and drawability and lower stiffness with respect to the iPB homopolymer. This mechanical behavior was correlated to phase transformations during deformation. In their initial, unstretched state, the samples with C2 content ≤7.6 mol % were crystallized in form II with a very low amount of form I′ crystals, whereas samples with higher ethylene contents were initially crystallized as a mixture of form II and form I′. During stretching, form II present in all the initial unoriented samples transforms into form I, indicating that uniaxial deformation significantly accelerates form II–form I transition. In quiescent conditions, in fact, this transformation typically required extended periods, with a time of half-transformation of form II into form I (*t*_1/2(II–I)_) ranging from 1.3 to 9 h depending on the copolymer composition. However, the effect of stretching on the form II–form I transition differs among the various copolymers. Specifically, both the critical strain at which the form II- form I transition begins (ε_c_) and the strain at which 50% of the initial form II transforms into form I (ε_0.5_) increase with increasing C2 content. At the highest explored strains, all the samples were basically crystallized in form I/I′, with the exception of sample C4C2–5.5, which still contained a significant amount of form II (approximately 32%) at ε = 500%. A quantitative analysis of WAXD data enabled the construction of a phase diagram for C4C2 copolymers, which identifies the regions of stability of the various polymorphic forms as a function of the ethylene content and deformation. In addition to the form II-to-I transition, crystallization of form I′ from the amorphous phase was observed during stretching for the samples containing 4.3, 5.5, and 16.3 mol % ethylene. Specifically, in the case of sample C4C2–4.3, after the yielding point and up to a strain of approximately 90%, only the transition from form II to form I takes place, and then, at higher strains, form I′ begins to crystallize from the amorphous phase, while the form II-to-form I transition continues. On contrast, in the case of the samples C4C2–5.5 and C4C2–16.3, crystallization of form I′ from amorphous phase already occurs at low deformation values.

Since structural evolution and mechanical properties are dynamically coupled during deformation, the in situ measurements combining structure and mechanical characterization techniques used in this work are crucial to elucidate structure–property relations in these systems. Also, the derived phase diagram is of great interest as it enables a correlation between the stability of the different polymorphic forms, the microstructural characteristics of the samples (i.e., ethylene content), and an external parameter (i.e., deformation).

## Supplementary Material



## Notes

Further experimental details; procedure to evaluate the degrees of crystallinity and the content of different crystalline phases from 1D diffraction patterns; ^13^C NMR spectra; table reporting mechanical parameters; kinetics of the form II–form I transition in samples C4C2–1.7, C4C2–4.3, and C4C2–5.5 under quiescent conditions; diffraction profiles of compression-molded films of C4C2–5.5 and C4C2–14.3 copolymers after aging at ambient temperature for 1 h; plots of mechanical parameters as a function of ethylene content; 1D diffraction profiles of the initial unoriented films of C4C2 copolymers used for X-ray diffraction measurements during tensile deformation; selected 2D WAXD patterns, and corresponding 1D diffraction profiles, recorded during the stretching of the samples C4C2–7.6, C4C2–9.1 and C4C2–16.3; comparison of degrees of crystallinity and fractions of the different crystalline phases in the copolymers as a function of strain; WAXD data recorded during the stretching of the iPB homopolymer; percentage of form I obtained from the transition of form II during stretching of the iPB homopolymer; 2D WAXD patterns recorded at the critical strain at which the form II–form I transition begins (ε_c_); procedure to calculate the degree of crystalline orientation of form II during stretching; Hermans’ orientation function as a function of strain; and fitted curves and extrapolation to 100% form I for the data reporting the percentage of global form I as a function of strain (PDF)
