# Supplementary material for: The Role of Stretching-Induced Phase Transformations in the Mechanical Properties of Isotactic 1‑Butene-ethylene Copolymers from Ziegler–Natta Catalyst
Source: Macromolecules. 2025 Dec 11;58(24):13252–66. doi: 10.1021/acs.macromol.5c01958 (PMC12752694; doi:10.1021/acs.macromol.5c01958)
Supplement: Supplementary file 1 [file ma5c01958_si_001.pdf]

The Role of Stretching-Induced Phase Transformations on the Mechanical Properties of Isotactic 1-Butene-Ethylene Copolymers from Ziegler-Natta Catalyst

Anna Malafronte, Rocco Di Girolamo, Angelo Giordano, Fabio De Stefano, Miriam Scoti, Claudio De Rosa**

Dipartimento di Scienze Chimiche, Università di Napoli Federico II, Complesso Monte S.Angelo, Via Cintia, 80126 Napoli, Italy.

SUPPORTING INFORMATION

Further experimental details

Characterization. ^{13}C NMR spectra were acquired in 1,1,2,2-tetrachloroethane-d_2 at 120 °C and recorded on a Bruker Avance III HD 400 spectrometer (^{1}H: 400 MHz, ^{13}C: 101 MHz). ^{13}C chemical shifts were referenced to the carbon signal of the deuterated solvent ($C_2D_2Cl_4$). BHT (0.5 mg/mL) was added as a stabilizer. From ^{13}C NMR spectra, the concentration of constitutional triads BBB, BBE, BEB, EBE, BEE and EEE (with B = 1-butene unit and E = ethylene unit) was determined.[S1,S2] Ethylene content in the copolymers was estimated from the concentration of the constitutional triads BEB, BEE, and EEE. The average lengths of butene and ethylene sequences were calculated according to Randall *et al.*[S2] using the following equations:

$$n(\mathrm{E}) = (\mathrm{EEE} + \mathrm{EEB} + \mathrm{BEB})/(\mathrm{BEB} + 0.5\mathrm{EEB})$$

$$n(\mathrm{B}) = (\mathrm{BBB} + \mathrm{BBE} + \mathrm{EBE})/(\mathrm{EBE} + 0.5\mathrm{BBE})$$

X-ray powder diffraction profiles of the unoriented compression molded films used for the study of mechanical properties and kinetics of transformation from form II into form I of samples C4C2-1.7, C4C2-4.3 and C4C2-5.5 in quiescent conditions were acquired at room temperature with Ni-filtered Cu Kα radiation (λ = 1.5418 Å) by using the Empyrean diffractometer by Panalytical with continuous scans of the 2θ angle and scanning rate of 0.02 degree/s.

Tensile tests. Unoriented compression molded films (thickness 0.3 – 0.5 mm) were prepared by melting samples at temperatures 30-40 °C higher than their melting temperature under a pressure lower than 5 bar, to avoid preferred orientations in the film, and cooling to room temperature at cooling rate of about 20 °C/min, by circulation of cold water in press plates. The mechanical tests were performed at room temperature on unoriented compression molded films with a universal mechanical tester Zwicky by Zwick Roell, following the standard test method ASTM D882. Rectangular specimens, 10 mm long and 5 mm width, cut from unoriented compression molded films were stretched up to the break. In the mechanical tests the ratio between the drawing rate and the initial length was fixed equal

to 0.1mm/(mm×min) for the measurement of Young's modulus and 10 mm/(mm×min) for the measurement of stress-strain curves and the determination of the other mechanical properties (stress and strain at yield and at break). The reported stress-strain curves and the values of the mechanical properties are averaged over at least five independent experiments.

*Kinetics of form II-form I transition in quiescent conditions in samples C4C2-1.7, C4C2-4.3 and C4C2-5.5.*The kinetics of transformation from form II into form I of samples C4C2-1.7, C4C2-4.3 and C4C2-5.5 was studied on melt-crystallized samples obtained by heating the samples up to 200 °C and cooling to 0 °C, at controlled rate of 10 °C/min. The samples were then heated to 25 °C at 10 °C/min and X-ray powder diffraction profiles of the samples were acquired at room temperature as soon as prepared (that is, as soon as after cooling from the melt to 0 °C and successive heating to 25 °C) (aging time $t_a = 0$) and after keeping the samples at room temperature for different aging time t_a.

Evaluation of the degrees of crystallinity and the content of different crystalline phases from 1D diffraction patterns

The degree of crystallinity (x_c) was determined from the 1D diffraction patterns by calculating the ratio between the area of the crystalline peaks (A_c) and the total area under the diffraction curve (A_t), according to the formula: $x_c = (A_c/A_t)\times 100$. To isolate A_c, the contribution of the amorphous phase was subtracted from A_t after proper normalization. For C4C2 copolymers containing up to 5.5 mol% ethylene, the amorphous halo was modeled using the diffraction pattern of atactic poly(1-butene). For copolymers with C2 content above 5.5 mol% ethylene, the amorphous contribution was instead obtained from the diffraction data of their molten counterparts.

We recall that form I of iPB crystallizes in two different modifications, showing the same diffraction profile. One is referred to as form I, which results from the transformation of form II, and the other is the low-melting form I', which forms either by direct crystallization from the melt or by crystallization

from the amorphous phase. Therefore, the percentage of crystals of total form I (form I + form I') ($f_{(\mathrm{I+I'})}$) with respect to the crystals of form II (f_{II}) was determined by the increase of the diffraction peak of the $(110)_{\mathrm{I}}$ reflection of form I at $q \approx 7.0$ nm^{-1} in 1D diffraction profiles by eq. S1: [S3,S4]

$$f_{(\mathrm{I+I'})} = \frac{A(110)_{\mathrm{I}}}{A(110)_{\mathrm{I}}+0.36\,A(200)_{\mathrm{II}}} \times 100 \qquad \text{(S1)}$$

where $A(110)_{\mathrm{I}}$ and $A(200)_{\mathrm{II}}$ are the diffraction areas of the reflections $(110)_{\mathrm{I}}$ of the trigonal form I (I') at $q \approx 7.0$ nm^{-1} and $(200)_{\mathrm{II}}$ of the tetragonal form II at $q \approx 8.4$ nm^{-1}, respectively. As a consequence, $f_{(\mathrm{I+I'})} + f_{\mathrm{II}} = 100$ and the fractions of crystals of total form I and form II relative to the entire mass of the sample are: $f'_{(\mathrm{I+I'})} = f_{(\mathrm{I+I'})} \times x_{\mathrm{c}}/100$ and $f'_{\mathrm{II}} = f_{\mathrm{II}} \times x_{\mathrm{c}}/100$, with $f'_{(\mathrm{I+I'})} + f'_{\mathrm{II}} = x_{\mathrm{c}}$.

Therefore, the values of the fractions of total form I and of form II with respect to the total mass of the samples ($f'_{(\mathrm{I+I'})}$ and f'_{II}, respectively) at the strain ε were calculated by eqs. S2 and S3:

$$f'_{(\mathrm{I+I'})}(\varepsilon) = f_{(\mathrm{I+I'})}(\varepsilon) \times x_{\mathrm{c}}(\varepsilon)/100 \qquad \text{(S2)}$$

$$f'_{\mathrm{II}}(\varepsilon) = f_{\mathrm{II}}(\varepsilon) \times x_{\mathrm{c}}(\varepsilon)/100 \qquad \text{(S3)}$$

with $f'_{(\mathrm{I+I'})}(\varepsilon) + f'_{\mathrm{II}}(\varepsilon) = x_{\mathrm{c}}(\varepsilon)$.

$f_{(\mathrm{I'+I})}$ corresponds to the percentage of crystals of total form I, that is low-melting form I' ($f_{\mathrm{I'}}$) *plus* high-melting form I (f_{I}).

In the case of the C4C2-1.7 sample, $f_{\mathrm{I'}} = 0$ and, therefore, the percentage of crystals of form I with respect to the crystals of form II evaluated from eq. S1 corresponds to the percentage of crystals of form I derived from transformation of form II into form I ($f_{(\mathrm{I+I'})} = f_{\mathrm{I}}$), and the fraction of total form I with respect to the total mass of the sample at the strain ε evaluated by eq. S2 corresponds to the fraction of form I obtained from form II- form I transformation ($f'_{(\mathrm{I+I'})}(\varepsilon) = f'_{\mathrm{I}}(\varepsilon)$).

The other samples exhibit a coexistence of form I' and form I crystals, which cannot be distinguished by X-ray diffraction, as both contribute to the $(110)_{\mathrm{I}}$ reflection of form I at $q \approx 7.0$ nm^{-1} in eq. S1. In these cases, the percentage of form I' was estimated by subtracting from the percentage of total form I

(i.e., form I' + form I) the percentage of form I determined from the decrease of the intensity peak at $q \approx 8.4$ nm^{-1} of form II during stretching. This approach is valid because the decrease in the peak at $q \approx 8.4$ nm^{-1} of form II is solely due to the form II-to-form I transition. In particular, the fractions of form I, with respect to the total mass of the sample, obtained from transformation of form II ($f'_{\mathrm{I(II)}}$) and of form I' obtained from crystallization of the amorphous phase ($f'_{\mathrm{I'(am)}}$), at the strain ε, are given by $f'_{\mathrm{I(II)}}(\varepsilon) = f'_{\mathrm{II}}(\varepsilon_0) - f'_{\mathrm{II}}(\varepsilon)$ and $f'_{\mathrm{I'(am)}}(\varepsilon) = f'_{\mathrm{(I+I')}}(\varepsilon) - f'_{\mathrm{I(II)}}(\varepsilon)$, respectively, and the fractions of the crystallinity x_c given by the crystals of form I obtained from transformation of form II ($f_{\mathrm{I(II)}}$) and by crystals of form I' obtained from crystallization of amorphous phase ($f_{\mathrm{I'(am)}}$), at the strain ε, were calculated by eqs. S4 and S5:

$$f_{\mathrm{I(II)}}(\varepsilon) = f'_{\mathrm{I(II)}}(\varepsilon)/x_{\mathrm{c}}(\varepsilon) \times 100 \qquad \text{(S4)}$$

$$f_{\mathrm{I'(am)}}(\varepsilon) = f'_{\mathrm{I'(am)}}(\varepsilon)/x_{\mathrm{c}}(\varepsilon) \times 100 \qquad \text{(S5)}$$

with $f_{\mathrm{(I+I')}}(\varepsilon) = f_{\mathrm{I(II)}}(\varepsilon) + f_{\mathrm{I'(am)}}(\varepsilon)$.

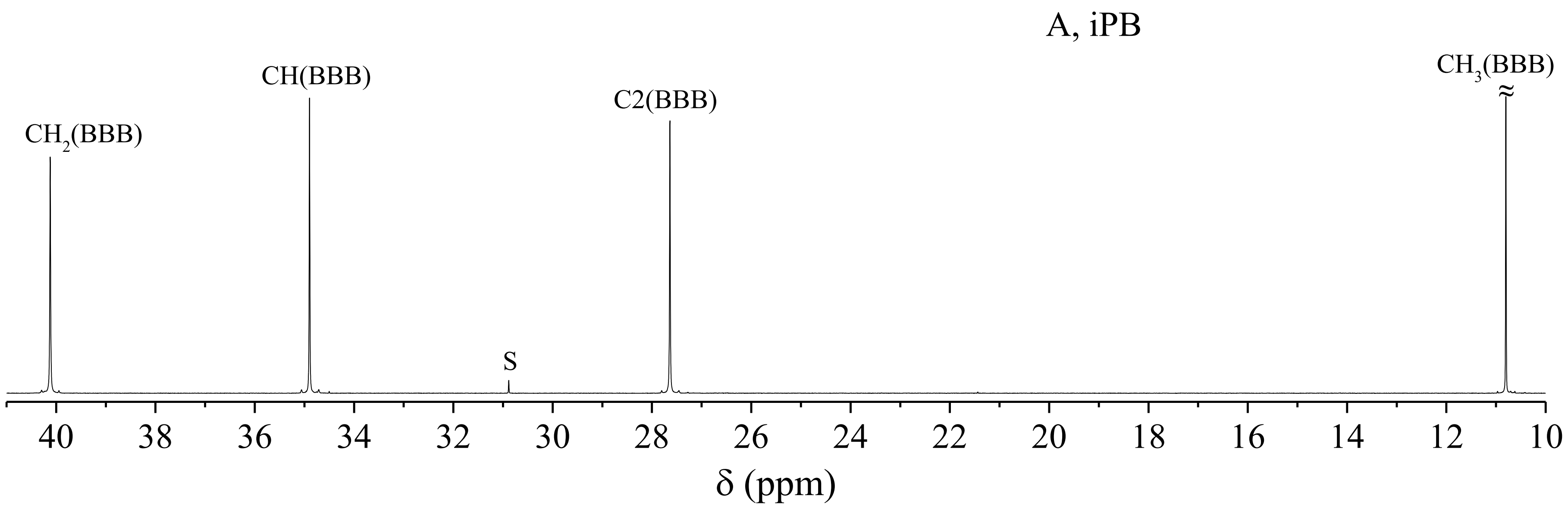
A, iPB
CH2(BBB)
CH(BBB)
S
C2(BBB)
CH3(BBB)
40
38
36
34
32
30
28
26
24
22
20
18
16
14
12
10
δ (ppm)

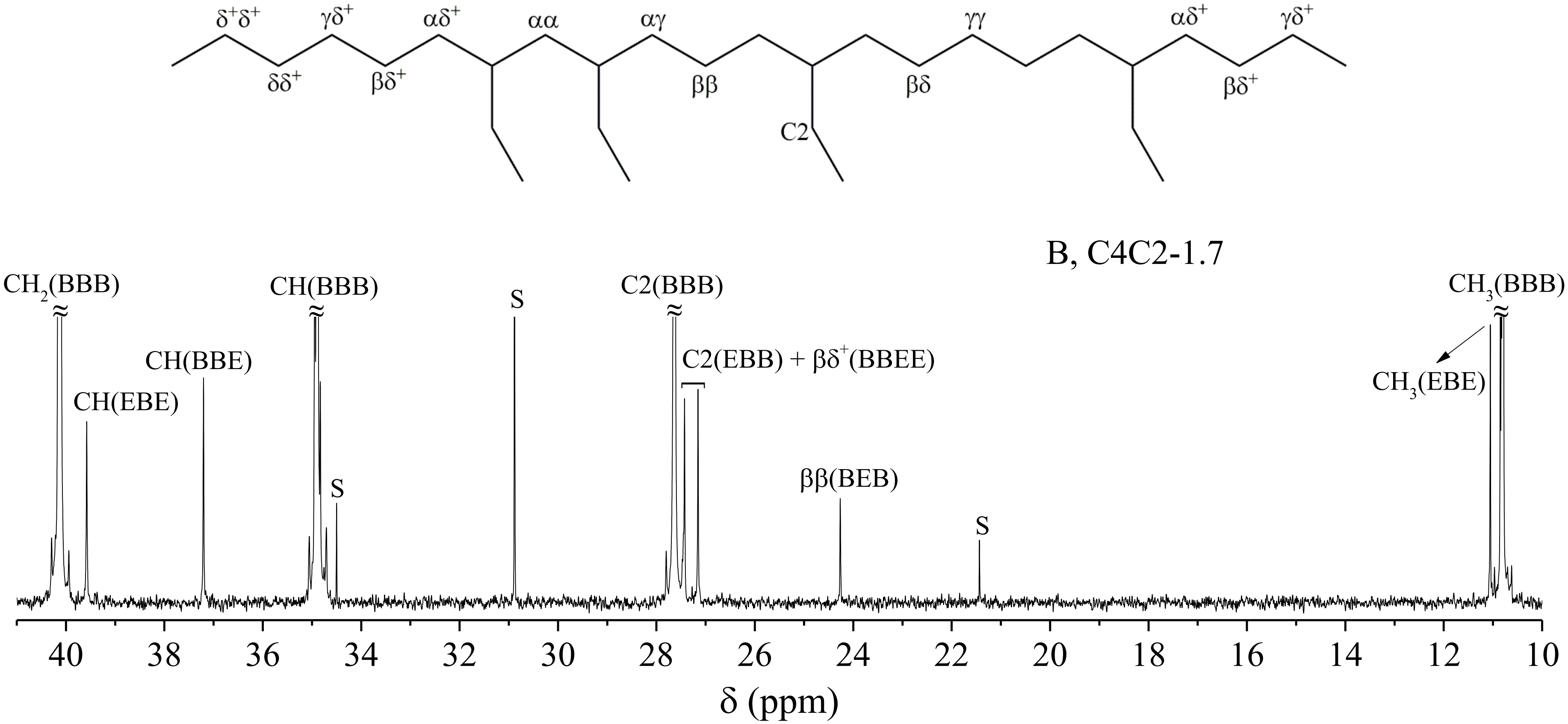

δ+δ+
γδ+
αδ+
αα
αγ
γγ
αδ+
γδ+
δδ+
βδ+
ββ
βδ
βδ+
C2
B, C4C2-1.7
CH2(BBB)
CH(EBE)
CH(BBE)
CH(BBB)
S
S
C2(BBB)
C2(EBB) + βδ+(BBEE)
ββ(BEB)
S
CH3(BBB)
CH3(EBE)
40 38 36 34 32 30 28 26 24 22 20 18 16 14 12 10
δ (ppm)

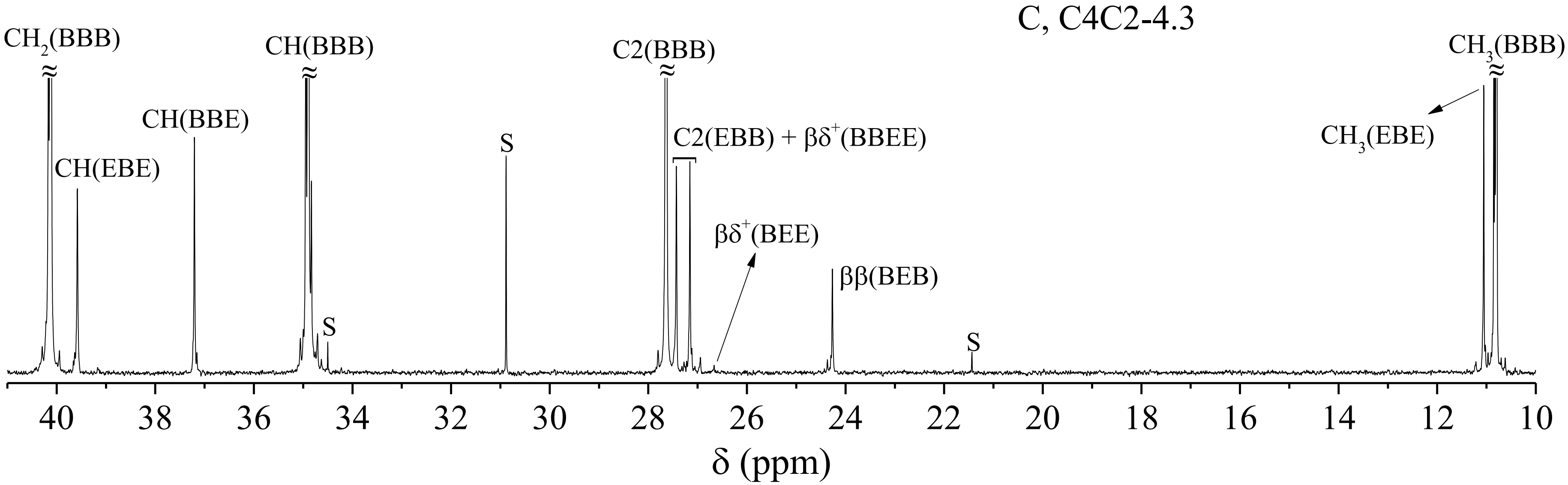
C, C4C2-4.3
CH2(BBB)
CH(BBB)
C2(BBB)
CH3(BBB)
CH(BBE)
CH(EBE)
S
C2(EBB) + βδ+(BBEE)
CH3(EBE)
βδ+(BEE)
ββ(BEB)
40 38 36 34 32 30 28 26 24 22 20 18 16 14 12 10
δ (ppm)

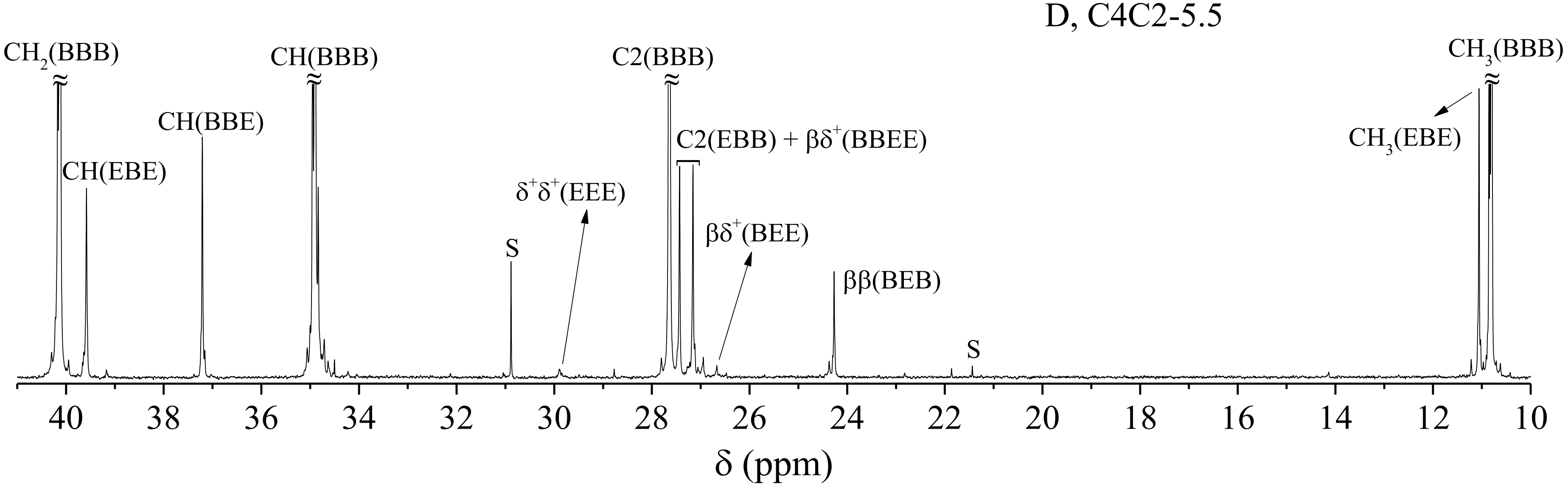
D, C4C2-5.5
CH2(BBB)
CH(BBB)
C2(BBB)
CH3(BBB)
CH(BBE)
CH(EBE)
C2(EBB) + βδ+(BBEE)
CH3(EBE)
δ+δ+(EEE)
βδ+(BEE)
S
ββ(BEB)
40 38 36 34 32 30 28 26 24 22 20 18 16 14 12 10
δ (ppm)

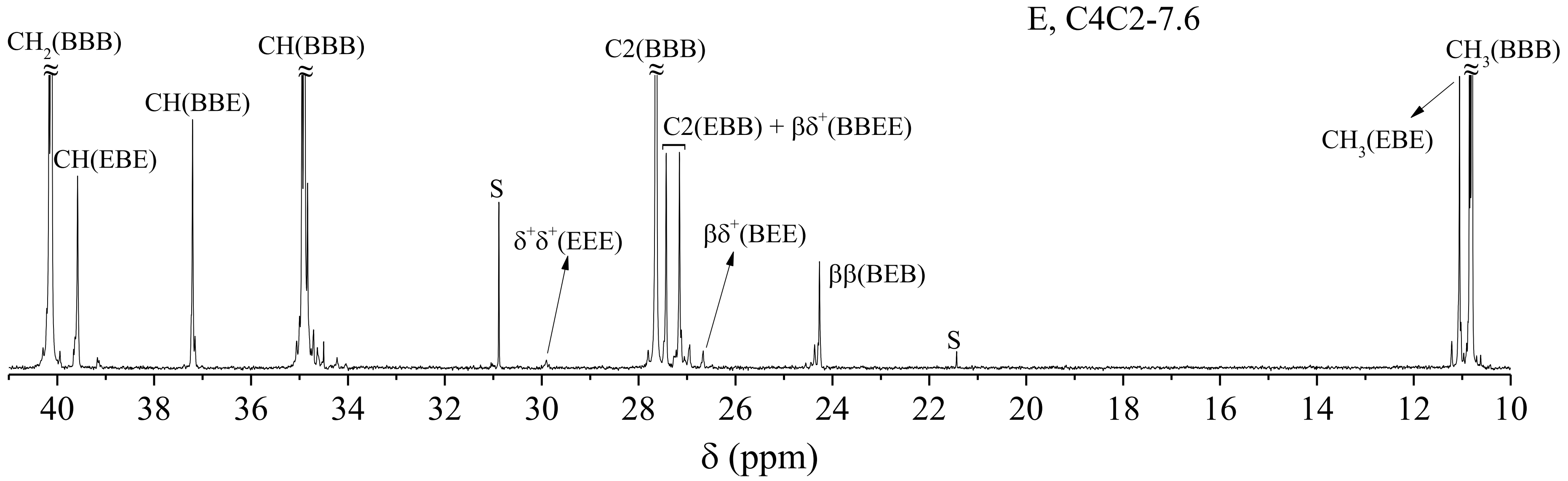
E, C4C2-7.6
CH2(BBB)
CH(EBE)
CH(BBE)
CH(BBB)
S
δ+δ+(EEE)
C2(BBB)
C2(EBB) + βδ+(BBEE)
βδ+(BEE)
ββ(BEB)
S
CH3(BBB)
CH3(EBE)
40 38 36 34 32 30 28 26 24 22 20 18 16 14 12 10
δ (ppm)

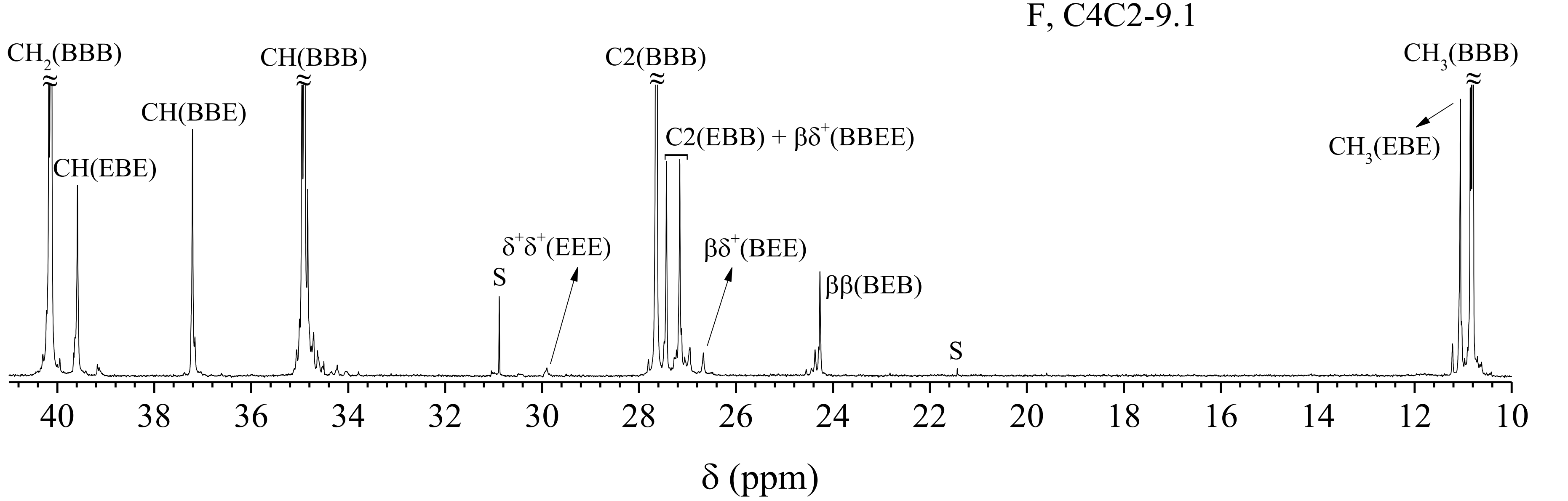
F, C4C2-9.1
CH2(BBB)
CH(EBE)
CH(BBE)
CH(BBB)
S
δ+δ+(EEE)
C2(BBB)
C2(EBB) + βδ+(BBEE)
βδ+(BEE)
ββ(BEB)
S
CH3(BBB)
CH3(EBE)
40 38 36 34 32 30 28 26 24 22 20 18 16 14 12 10
δ (ppm)

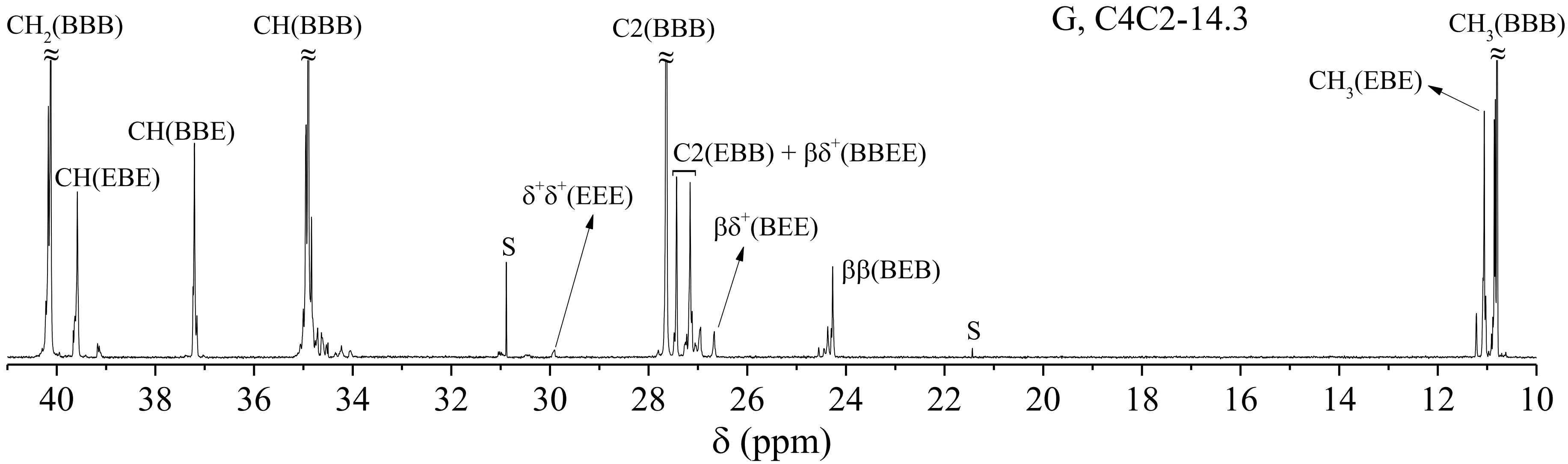

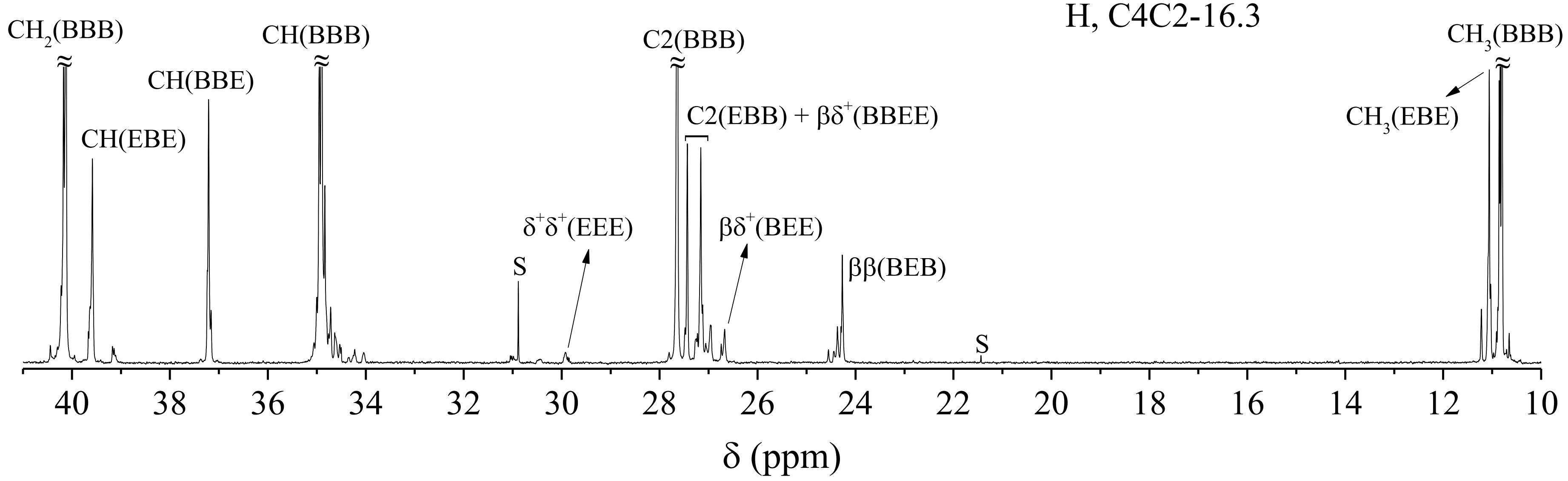

Figure S1. ^{13}C NMR spectra of the iPB homopolymer (A) and the copolymers C4C2-1.7 (B), C4C2-4.3 (C), C4C2-5.5 (D), C4C2-7.6 (E), C4C2-9.1 (F), C4C2-14.3 (G), and C4C2-16.3 (H). A representative butene-ethylene sequence and the corresponding nomenclature

used for assigning the carbon atoms are shown in (B). S indicates the signals arising from BHT, which was used as stabilizer during the high-temperature NMR acquisition (120 °C).

Table S1. Ethylene (C2) content, concentrations of the comonomeric sequences BBB, BBE, EBE, BEB, BEE, and EEE (with B = 1-butene unit and E = ethylene unit), and average lengths of the E and B sequences, *n*(E) and *n*(B), in the C4C2 copolymer determined by ^{13}C NMR.

Sample	C2 (mol %)[a]	BBB (%)	BBE (%)	EBE (%)	BEB (%)	BEE (%)	EEE (%)	*n*(E)	*n*(B)
C4C2-1.7	1.7	91.5	7.9	0.1	1.7	<0.1	<0.1	1.0	24.6
C4C2-4.3	4.3	87.3	8.1	0.3	4.3	0.1	<0.1	1.01	22.00
C4C2-5.5	5.5	83.1	10.8	0.6	5.2	0.1	0.2	1.05	15.75
C4C2-7.6	7.6	79.33	11.86	1.26	6.29	0.95	0.31	1.12	12.85
C4C2-9.1	9.1	76.03	13.30	1.56	7.23	1.51	0.37	1.14	11.07
C4C2-14.3	14.3	63.53	19.59	2.59	10.97	2.56	0.75	1.17	6.92
C4C2-16.3	16.3	59.16	20.75	3.79	12.08	3.22	0.99	1.19	5.91

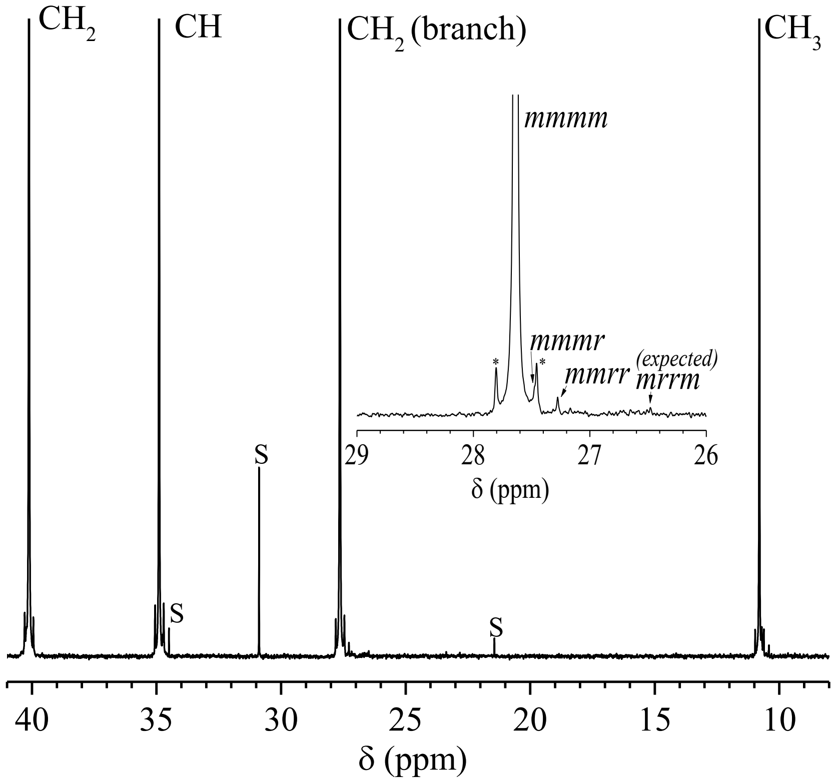

Figure S2. ^{13}C NMR spectrum of the iPB homopolymer synthesized with the same catalyst and under the same conditions used for the C4C2 copolymers. The inset shows the CH_2 branch region and the pentad assignments *Peaks due to ^{13}C-^{13}C coupling.

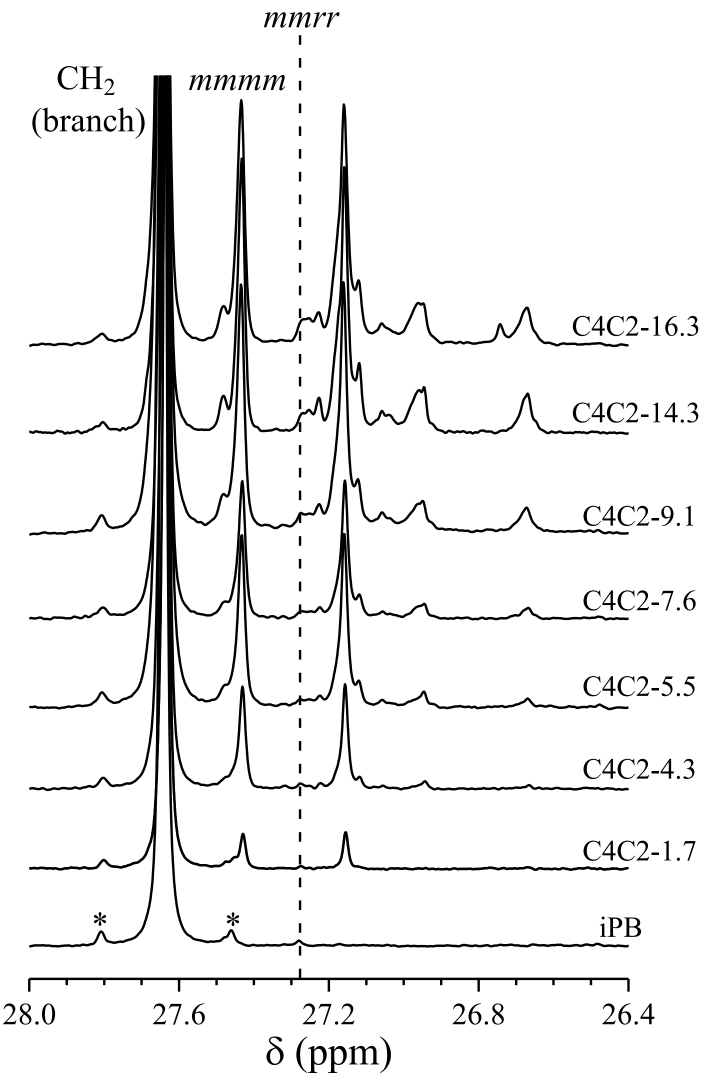

Figure S3. Comparison of the CH_2 branch region of the ^{13}C NMR spectra of the iPB homopolymer and C4C2 copolymers. The relative intensity of the NMR signal associated with the mmrr pentad in the spectra of the copolymers remains essentially unchanged compared with its relative intensity in the spectrum of the iPB homopolymer, indicating that the isotacticity of the homopolymer is likely retained in the copolymers. *Peaks due to ^{13}C-^{13}C coupling.

Table S2. Averaged values of Young's modulus (*E*), stress and strain at yielding (σ_y and ε_y) and stress and strain breaking (σ_b and ε_b) of compression molded films of C4C2 copolymers, immediately after preparation (fresh samples, Figure 1A) and after prolonged aging at ambient temperature (aged samples, Figure 1B). The degrees of crystallinity (x_c) and the percentages of crystals of form I' obtained from crystallization of amorphous ($f_{I'}$), form I obtained from transition of form II (f_I) and form II (f_{II}) are reported.

Sample	C2 (mol%)	*E* (MPa)	σ_y (MPa)	ε_y (%)	σ_b (MPa)	ε_b (%)	x_c (%)	Crystal forms of iPB	$f_{I'}$ (%)	f_I (%)	f_{II} (%)
**Fresh samples**											
C4C2-1.7	1.7	81 ± 5	6.2 ± 0.4	6 ± 3	30 ± 6	410 ± 30	60	II + I'	4	-	96
C4C2-4.3	4.3	45 ± 2	4.3 ± 0.3	15 ± 3	16 ± 3	360 ± 40	47	II + I'	4	-	96
C4C2-5.5	5.5						18	II	0	-	100
C4C2-5.5 t_a=1h[a]	5.5	40 ± 3	2.10 ± 0.15	15 ± 2	14 ± 4	335 ± 80	42	II	0	-	100
C4C2-7.6	7.6	35 ± 2	2.2 ± 0.5	20 ± 5	25 ± 2	760 ± 20	32	II + I'	5	-	95
C4C2-9.1	9.1	30 ± 2	2.0 ± 0.3	25 ± 1	22 ± 1	1100 ± 90	30	II + I'	40	-	60
C4C2-14.3 t_a=1h[a]	14.3	17 ± 3	1.4 ± 0.2	22 ± 5	11 ± 1	1300 ± 90	15	I'	100	-	0
C4C2-16.3	16.3	10 ± 1	1.2 ± 0.1	22 ± 2	12 ± 2	890 ± 30	<5	I' + II + III	n.d.	-	n.d.
**Aged samples**											
C4C2-1.7	1.7	176 ± 9	17.0 ± 0.4	20 ± 2	30 ± 3	410 ± 45	63	I + I' + II	4	93	3
C4C2-4.3	4.3	123 ± 9	10 ± 1	24 ± 4	20 ± 2	430 ± 30	53	I + I'	4	96	0
C4C2-5.5	5.5	102 ± 3	8.0 ± 0.5	25 ± 5	20 ± 1	360 ± 30	45	I	0	100	0
C4C2-7.6	7.6	80 ± 8	6 ± 1	28 ± 1	27 ± 4	850 ± 6	43	I + I'	50	50	0
C4C2-9.1	9.1	63 ± 5	5.4 ± 0.5	30 ± 2	22 ± 1	1000 ± 60	42	I + I'	60	40	0
C4C2-14.3	14.3	30 ± 3	3.0 ± 0.4	27 ± 4	12 ± 1	1300 ± 30	28	I'	100	0	0
C4C2-16.3	16.3	35 ± 5	1.7 ± 0.2	20 ± 1	11 ± 1	690 ± 20	32	I' + III	n.d.	n.d.	n.d.

[a]) compression molded films aged at ambient temperature for 1 h (Figure S6). n.d. = not determined

Kinetics of form II-form I transition in quiescent conditions in samples C4C2-1.7, C4C2-4.3 and C4C2-5.5.

The transformation kinetics from form II to form I for samples C4C2-1.7, C4C2-4.3 and C4C2-5.5 was investigated by recording X-ray powder diffraction patterns of melt-crystallized specimens aged at ambient temperature for various aging times (t_a). The percentage of form I crystals produced from the conversion of form II (f_I), relative to the amount of form II, at different aging times, was calculated using eq. S1, and is shown in Figure S4. This is compared with the transformation kinetics of the iPB homopolymer as reported in Ref. S5. The time of half-transformation of form II into form I (i.e., the aging time at which 50% of the initial form II transforms into form I), indicated as $t_{1/2\ (II\text{-}I)}$, was found to be approximately 23 h for the iPB homopolymer, and 9 h and 2.5 h for the samples C4C2-1.7 and C4C2-4.3, respectively (Figure S4).

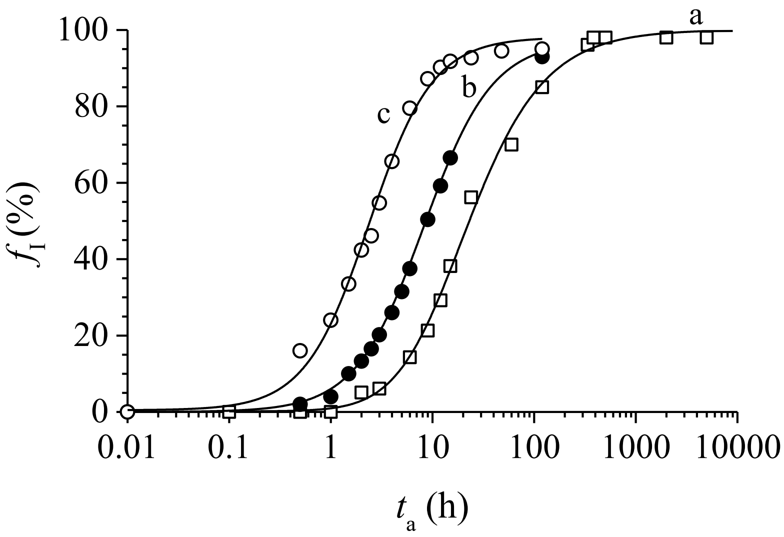

Figure S4 Percentage of crystals of form I obtained from transformation of form II with respect to the crystals of form II (f_I) at different values of aging time (t_a) for the iPB homopolymer (opened squares, curve a)[S5] and melt-crystallized C4C2-1.7 (filled circles, curve b) and C4C2-4.3 (opened circles, curve c) samples.

X-ray powder diffraction patterns of the melt-crystallized C4C2-5.5 sample during aging are displayed in Figure S5A. The sample C4C2-5.5 crystallizes directly into form II upon melt cooling, with an initial crystallinity of 18% (profile a in Figure S5A and Table S2). Aging at ambient temperature of this sample induces the transformation of form II to form I (profile c in Figure 1B) and leads to a marked increase in crystallinity from 18% to 45% (Table S2). The data in Figure S5A reveal that the increase in crystallinity (from 18% to 38-42%) occurs during the early stages of aging (t_a = 0.5-1 h) and is linked to further crystallization of form II from the amorphous phase. This is indicated by the rising intensity of the $(200)_{II}$ reflection at $2\theta \approx 12°$ and the absence of the $(110)_{I}$ reflection at $2\theta \approx 9.9°$ in profiles b and c of Figure S5A. After about 2 hours, the conversion from form II to form I begins, as evidenced by the appearance of the $(110)_{I}$ peak at $2\theta \approx 10°$ in profile d of Figure S5A. The intensity of this reflection continues to grow over time, while the $(200)_{II}$ reflection weakens, and overall crystallinity remains stable (profiles e-f of Figure S5A). The percentage of form I crystals produced from form II, calculated via eq. S1, is shown in Figure S5B. The time of half-transformation of form II into form I ($t_{1/2\ (II\text{-}I)}$) results equal to 4h.

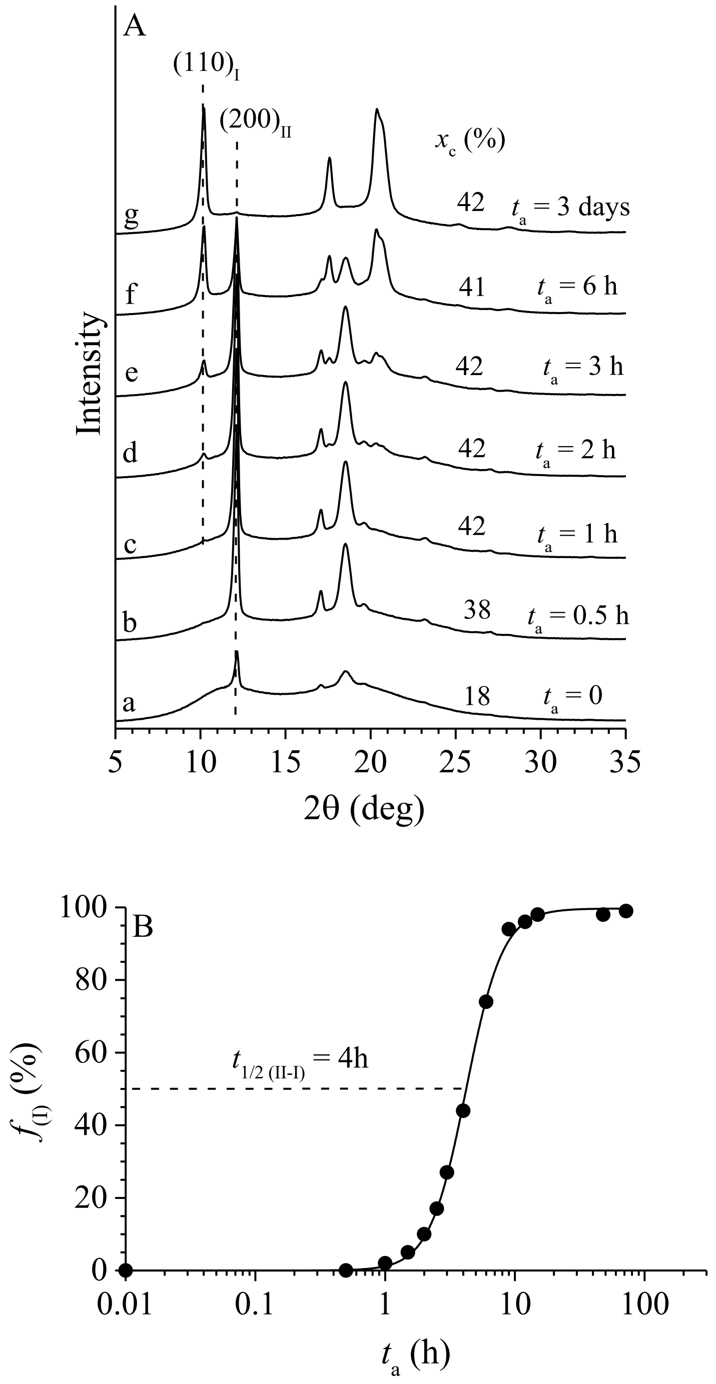

Figure S5. Some selected X-ray powder diffraction profiles recorded during aging at room temperature (A) and percentage of crystals of form I (f_I) with respect to the crystals of form II at different values of aging time t_a (B) of melt-crystallized C4C2-5.5 copolymer. In A, aging times (t_a) and degrees of crystallinity x_c at the various aging times are indicated in A.

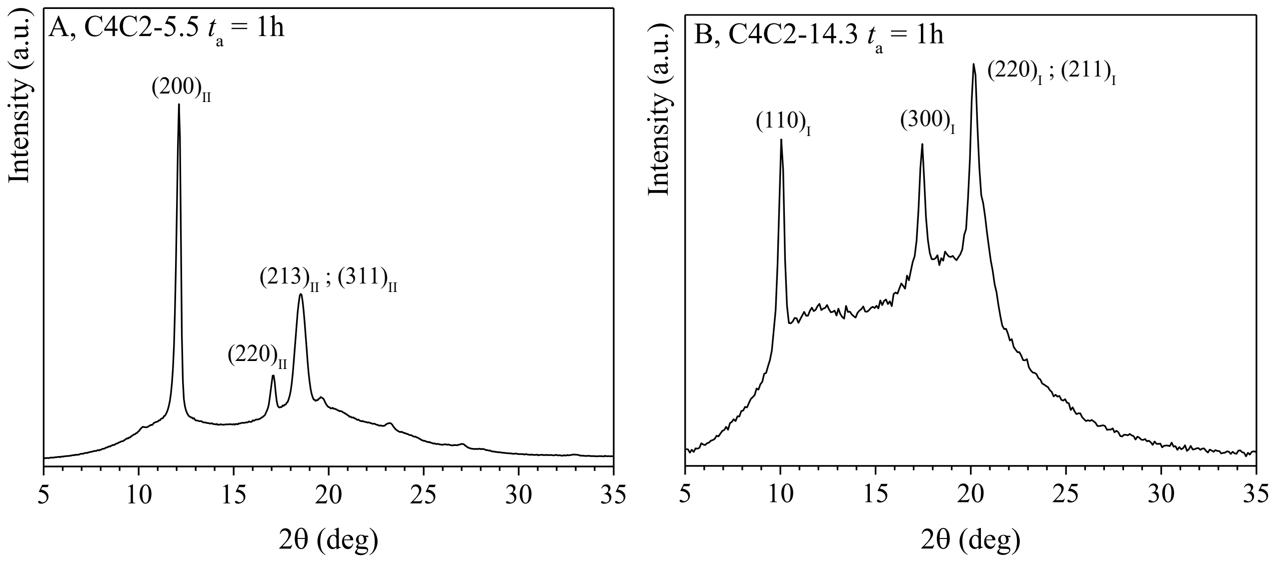

Figure S6. Diffraction profiles of compression molded films of C4C2-5.5 (A) and C4C2-14.3 (B) copolymers after aging at room temperature for t_a = 1h.

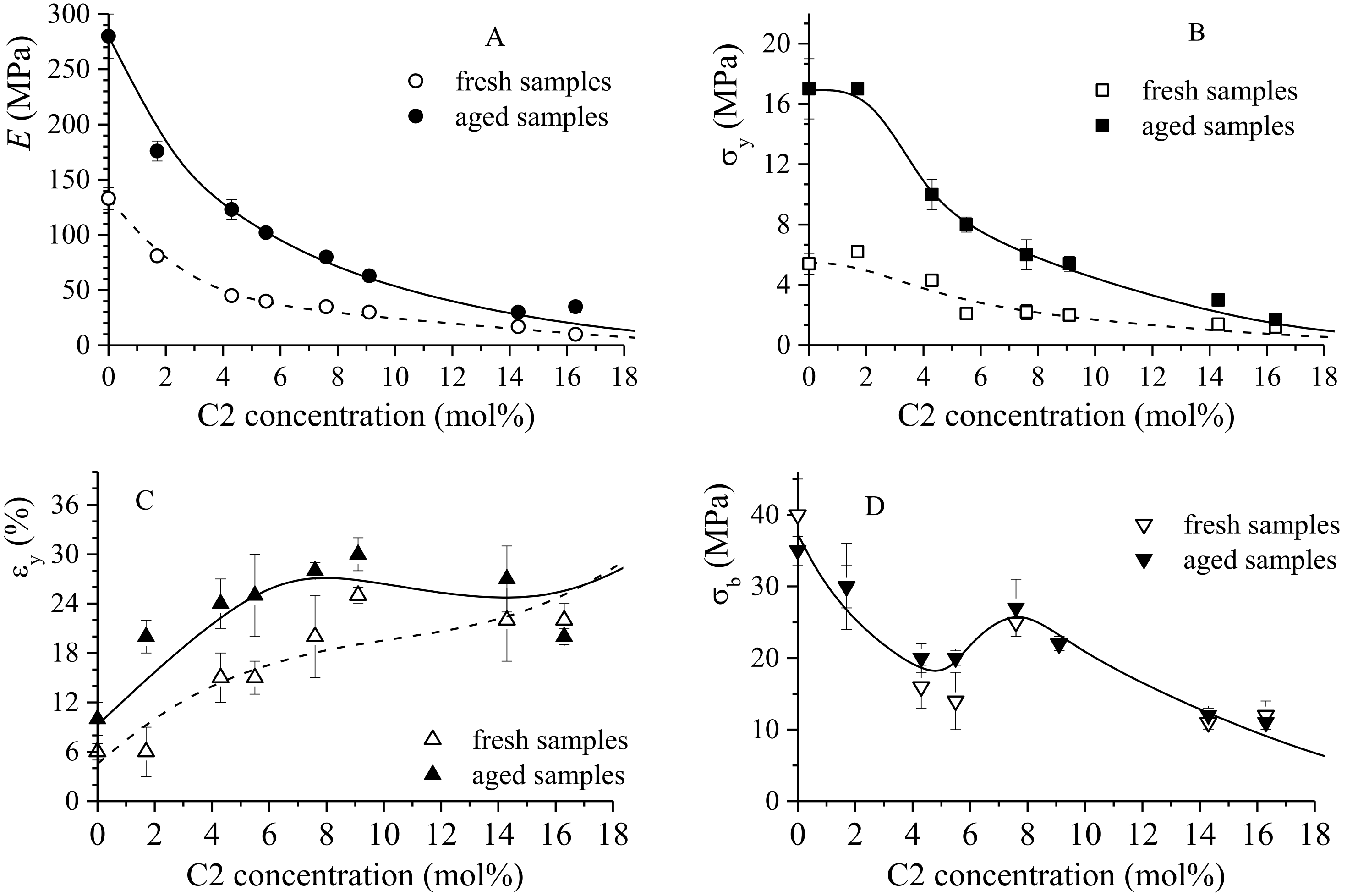
A
E (MPa)
fresh samples
aged samples
C2 concentration (mol%)
B
σy (MPa)
fresh samples
aged samples
C2 concentration (mol%)
C
εy (%)
fresh samples
aged samples
C2 concentration (mol%)
D
σb (MPa)
fresh samples
aged samples
C2 concentration (mol%)

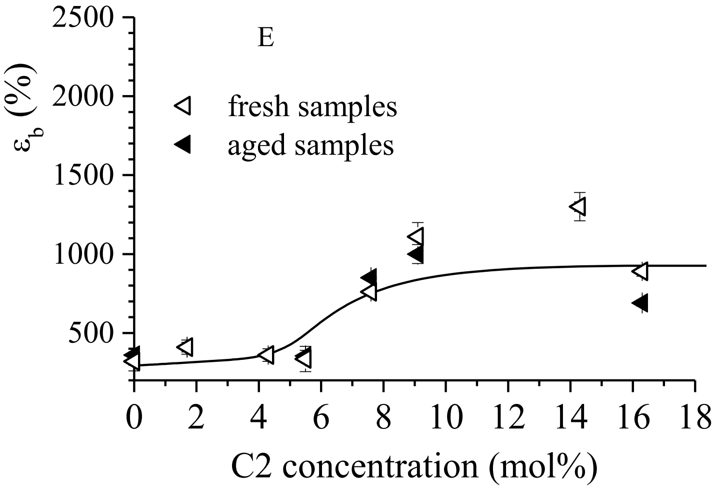

....

Figure S7. Averaged values of Young's modulus E (A), stress σ_y (B) and strain ε_y (C) at yielding, and stress σ_b (D) and strain ε_b (E) at break of compression molded films of C4C2 copolymers as soon as prepared (open black symbols) and after long aging times at room temperature (solid black symbols), as a function of the ethylene content evaluated from stress-strain curves reported in Figure 2A and 2B, respectively. Mechanical parameters obtained in the case of an iPB homopolymer synthetized with the same catalytic system used for the C4C2 copolymers are added for comparison.[S6]

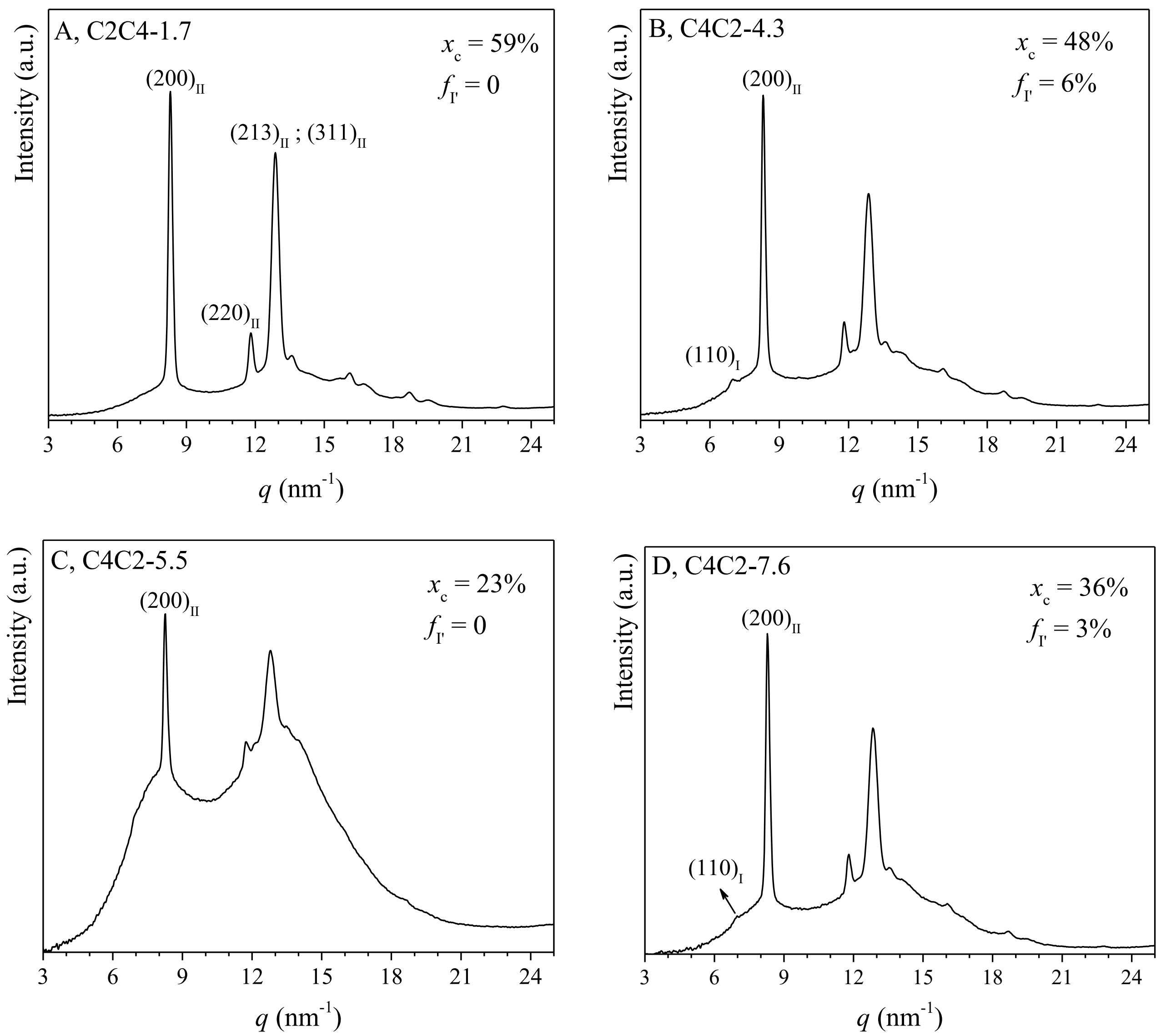
A, C2C4-1.7
x_c = 59%
$f_{I'}$ = 0
$(200)_{II}$
$(213)_{II}$; $(311)_{II}$
$(220)_{II}$
B, C4C2-4.3
x_c = 48%
$f_{I'}$ = 6%
$(200)_{II}$
$(110)_{I}$
C, C4C2-5.5
x_c = 23%
$f_{I'}$ = 0
$(200)_{II}$
D, C4C2-7.6
x_c = 36%
$f_{I'}$ = 3%
$(200)_{II}$
$(110)_{I}$
Intensity (a.u.)
q (nm^{-1})
3
6
9
12
15
18
21
24

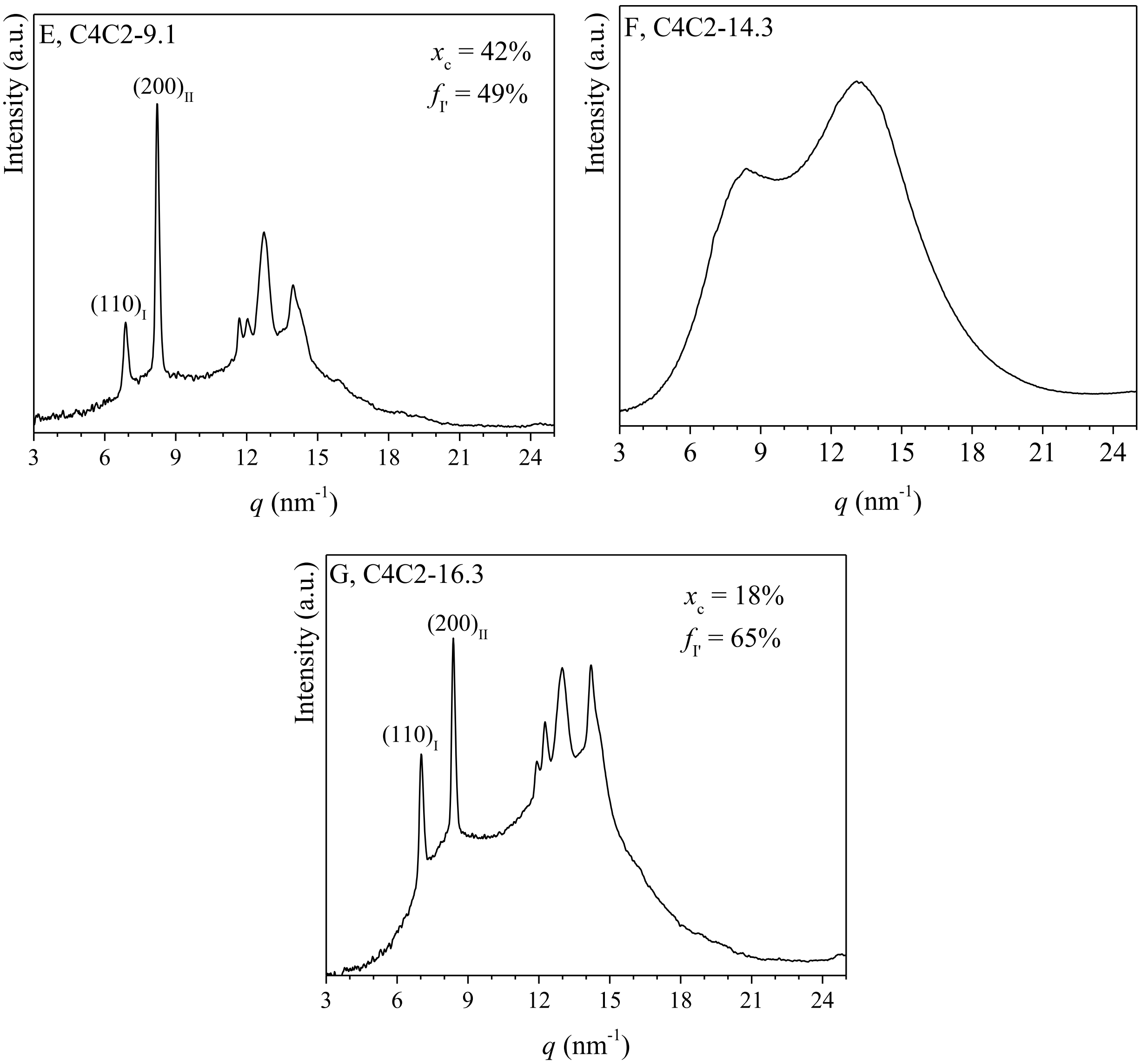

Figure S8. 1D diffraction profiles of the initial unoriented films of C4C2 copolymers used for X-ray diffraction measurements during tensile deformation. Profiles of the samples with ethylene content ≤ 14.3 mol% (A-F) were acquired as soon after cooling from the melt at room temperature, while profile of the sample C2C4-16.3 (G) was acquired after aging the film at room temperature for ≈ 2 h. Degrees of crystallinity (x_c) and percentage of crystals of form I' with respect to the crystals of form II ($f_{I'}$) are indicated.

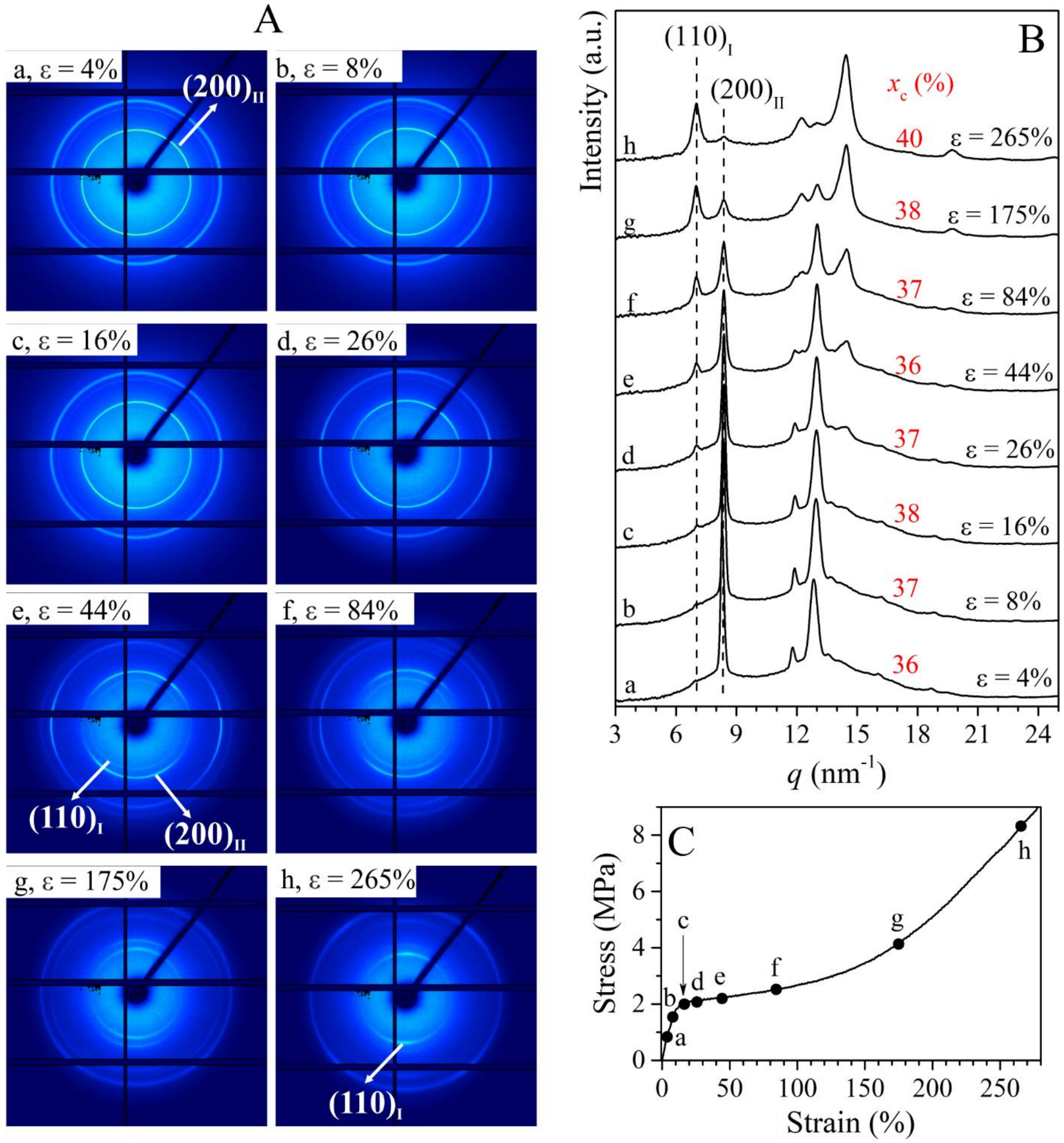

Figure S9. Selected 2D WAXD patterns (A), and corresponding 1D diffraction profile integrated over the coordinate azimuthal χ (B), recorded during the stretching of the sample C4C2-7.6 with 7.6 mol% of ethylene at the indicated values of strain ε, corresponding to the points a–h of the stress-strain curve (C). The tensile stretching axis lies along the 0-180° (horizontal) direction of the 2D WAXS patterns. In B, the degrees of crystallinity x_c are indicated.

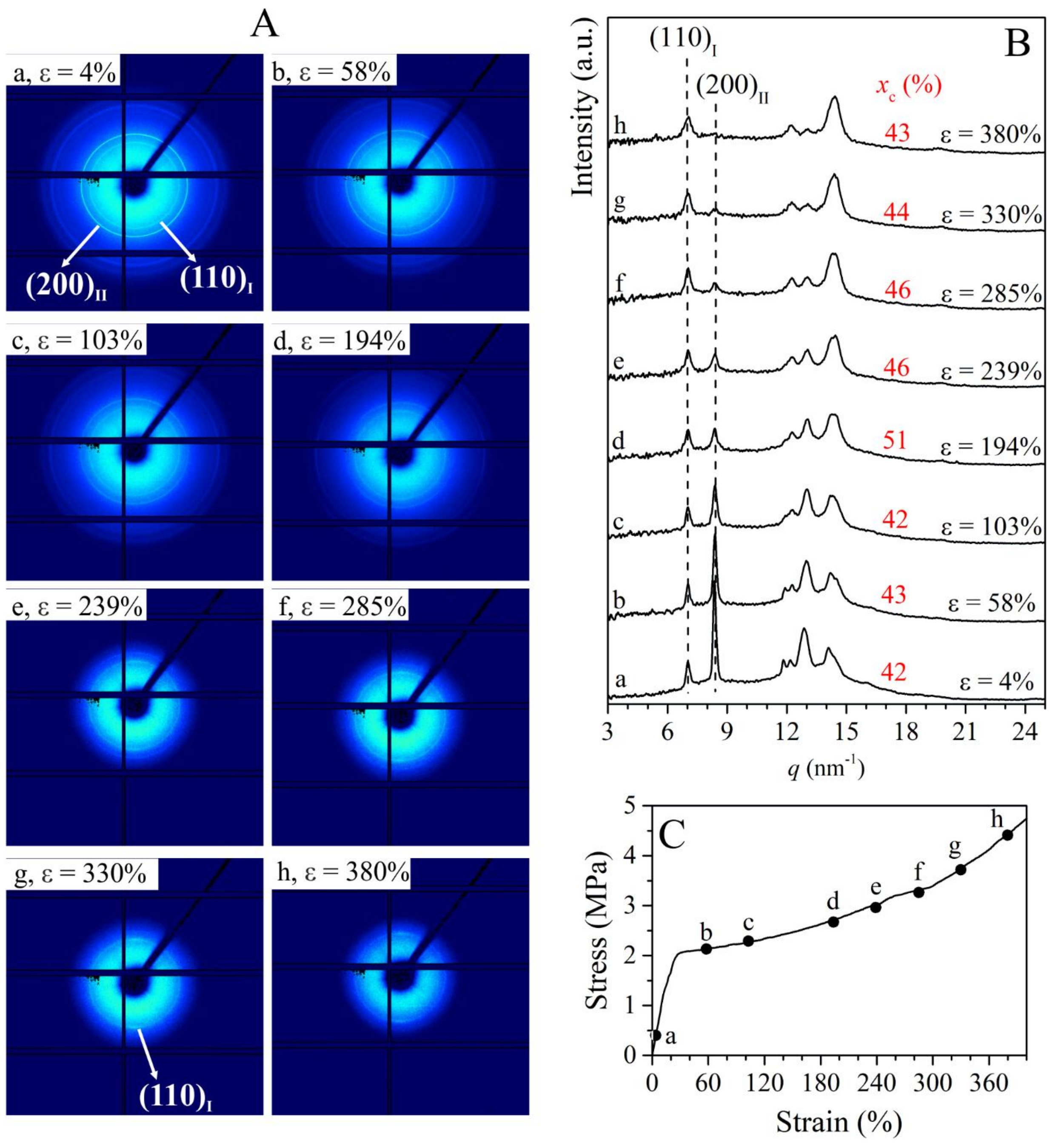

Figure S10. Selected 2D WAXD patterns (A), and corresponding 1D diffraction profile integrated over the coordinate azimuthal χ (B), recorded during the stretching of the sample C4C2-9.1 with 9.1 mol% of ethylene at the indicated values of strain ε, corresponding to the points a–h of the stress-strain curve (C). The tensile stretching axis lies along the 0-180° (horizontal) direction of the 2D WAXS patterns. In B, the degrees of crystallinity x_c are indicated.

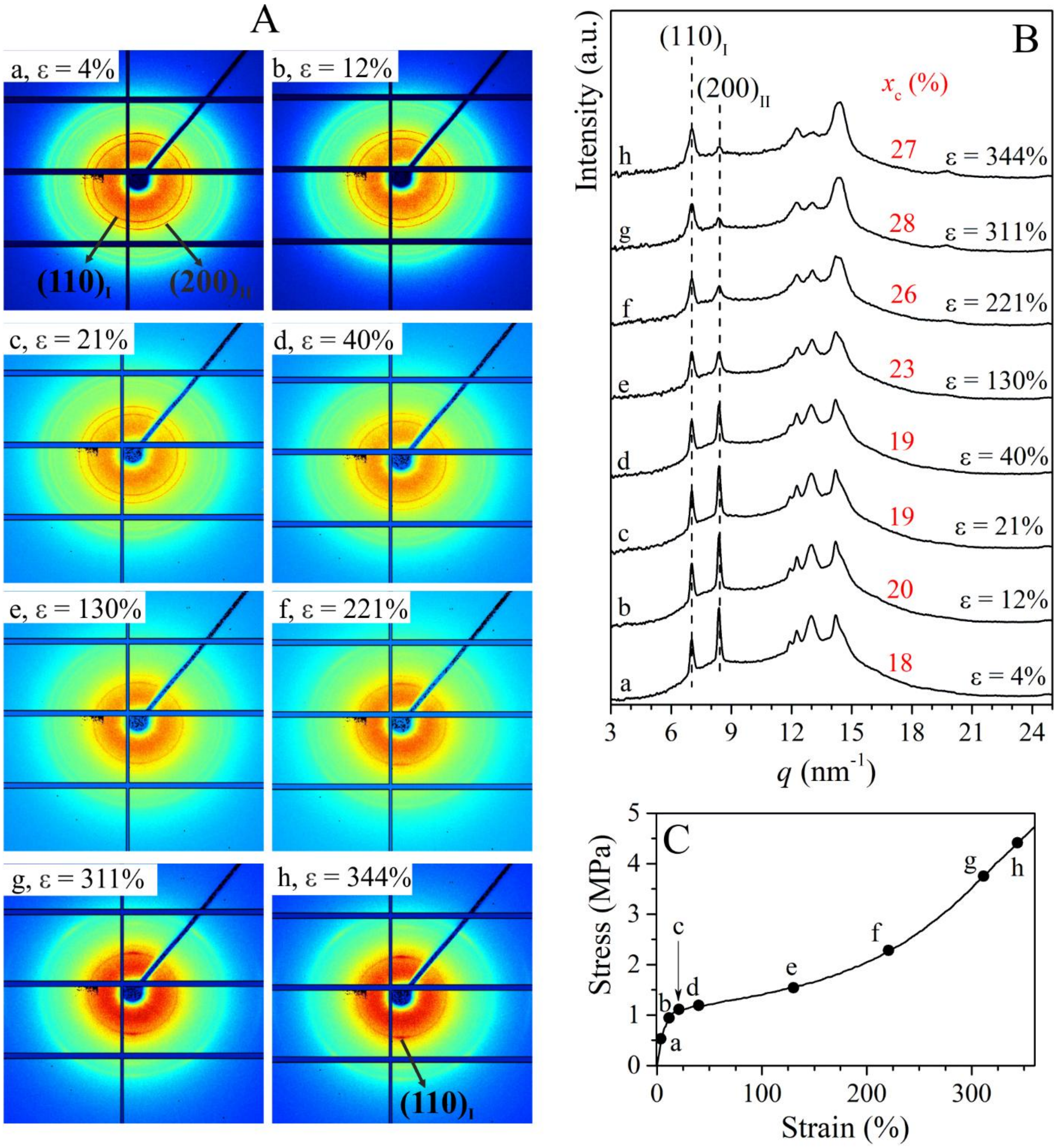

Figure S11. Selected 2D WAXD patterns (A), and corresponding 1D diffraction profile integrated over the coordinate azimuthal χ (B), recorded during the stretching of the sample C4C2-16.3 with 16.3 mol% of ethylene at the indicated values of strain ε, corresponding to the points a–h of the stress-strain curve (C). The tensile stretching axis lies along the 0-180° (horizontal) direction of the 2D WAXS patterns. In B, the degrees of crystallinity x_c are indicated.

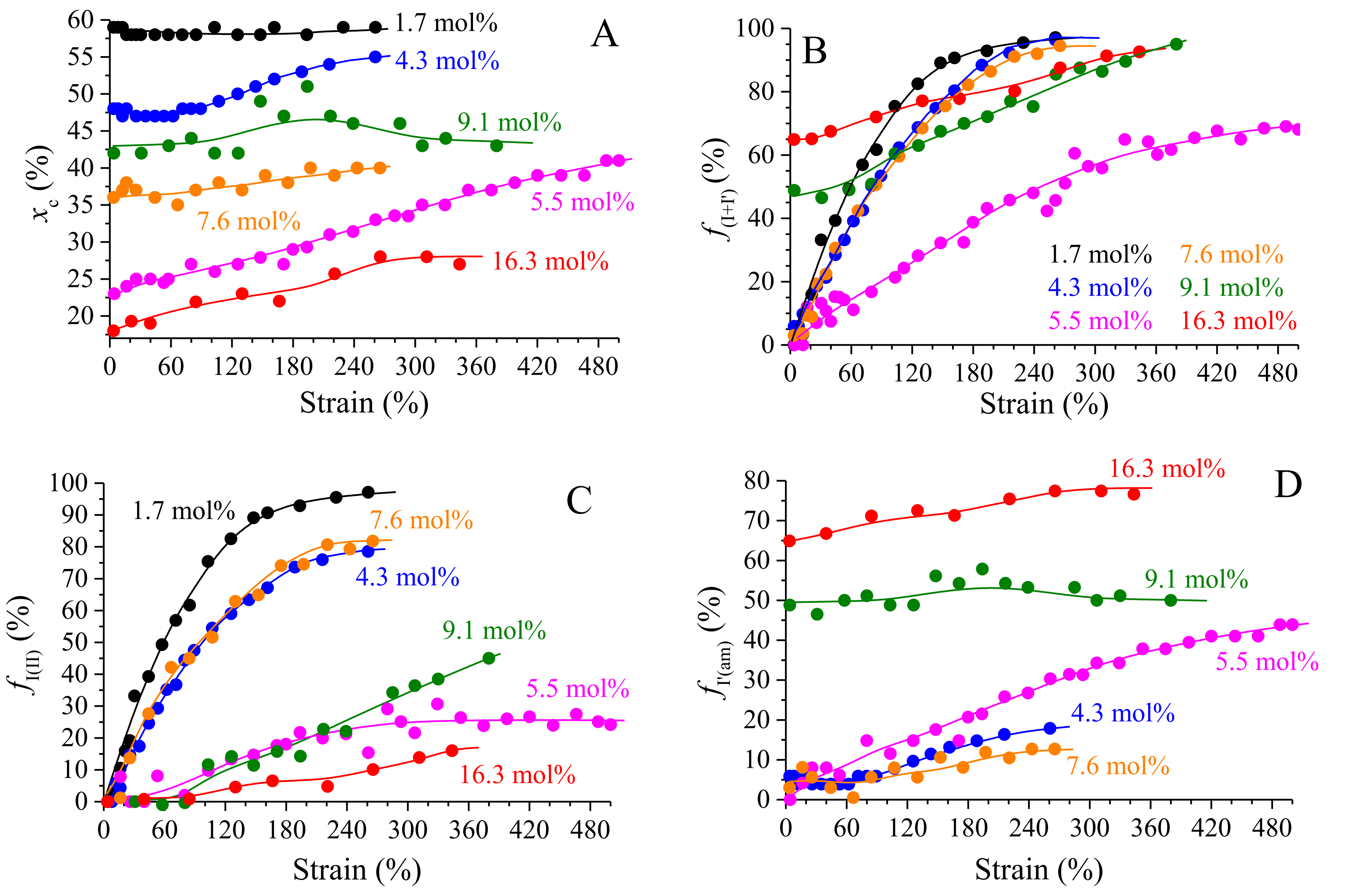
A
B
C
D
x_c (%)
$f_{(I+I')}$ (%)
$f_{I(II)}$ (%)
$f_{I'(am)}$ (%)
Strain (%)
1.7 mol%
4.3 mol%
5.5 mol%
7.6 mol%
9.1 mol%
16.3 mol%

Figure S12. Degrees of crystallinity (x_c, A), fractions of the crystallinity given by crystals of global form I ($f_{(I+I')}$, B), form I originated from transition of form II ($f_{I(II)}$, C) and form I' obtained from crystallization of the amorphous phase ($f_{I'(am)}$, D) for the samples C4C2-1.7 (black), C4C2-4.3 (blue), C4C2-5.5 (magenta), C4C2-7.6 (orange), C4C2-9.1 (olive), and C4C2-16.3 (red) as a function of strain. The ethylene content in each sample is indicated. For the sample C4C2-1.7 (black), $f_{(I+I')} = f_{I(II)}$ and $f_{I'(am)} = 0$.

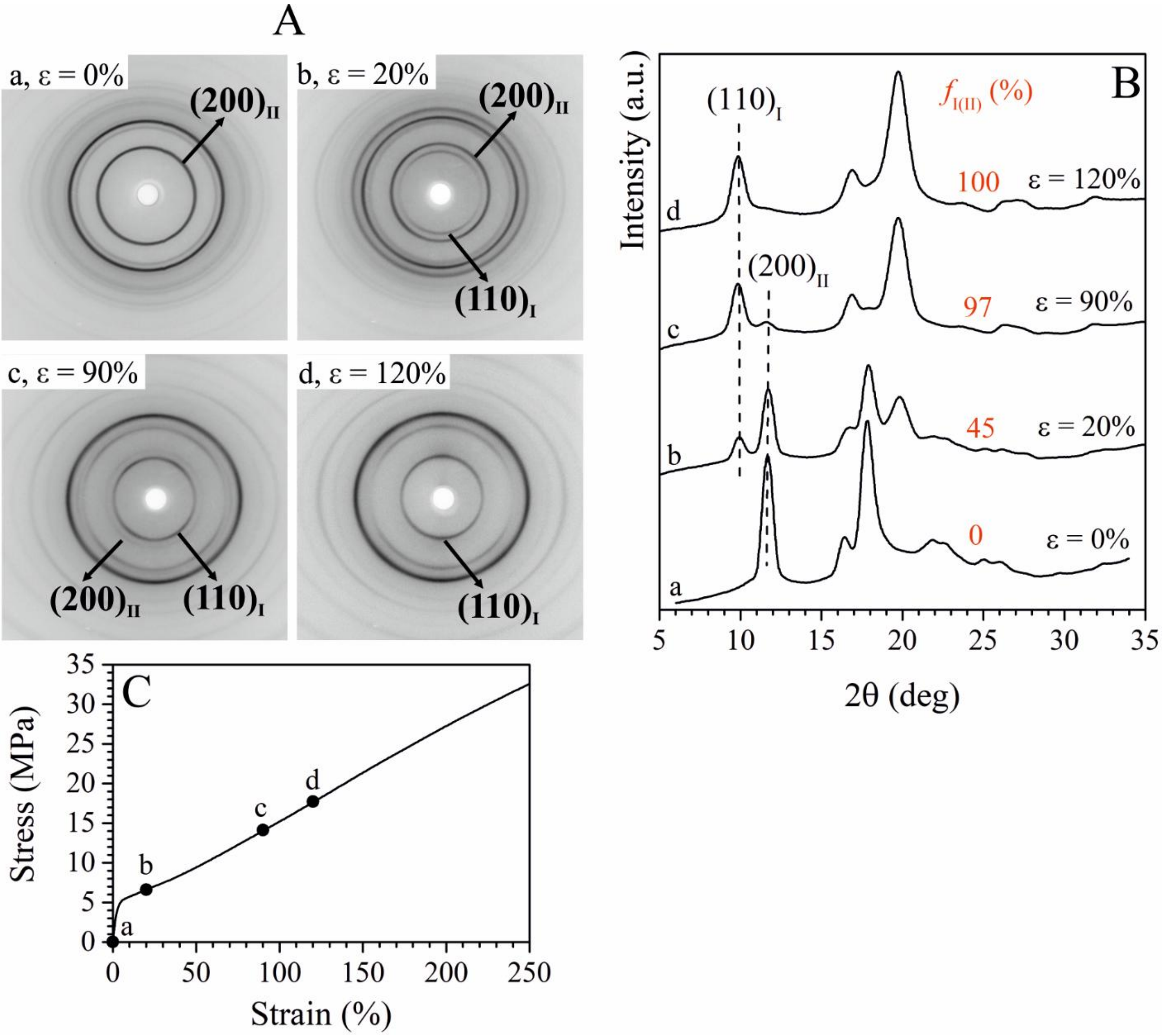

Figure S13. Selected 2D WAXD patterns (A), and corresponding 1D diffraction profile integrated over the azimuthal coordinate χ (B), recorded during the stretching of an isotactic poly(1-butene) (iPB) homopolymer, synthesized using the same catalytic system as that employed for the C4C2 copolymers, at the indicated values of strain ε, corresponding to the points a-d on the stress-strain curve (C). The tensile stretching axis lies along the 0-180° (horizontal) direction of the 2D WAXS patterns. In B, the percentages of form I originating from transition of form II ($f_{I(II)}$) are indicated.

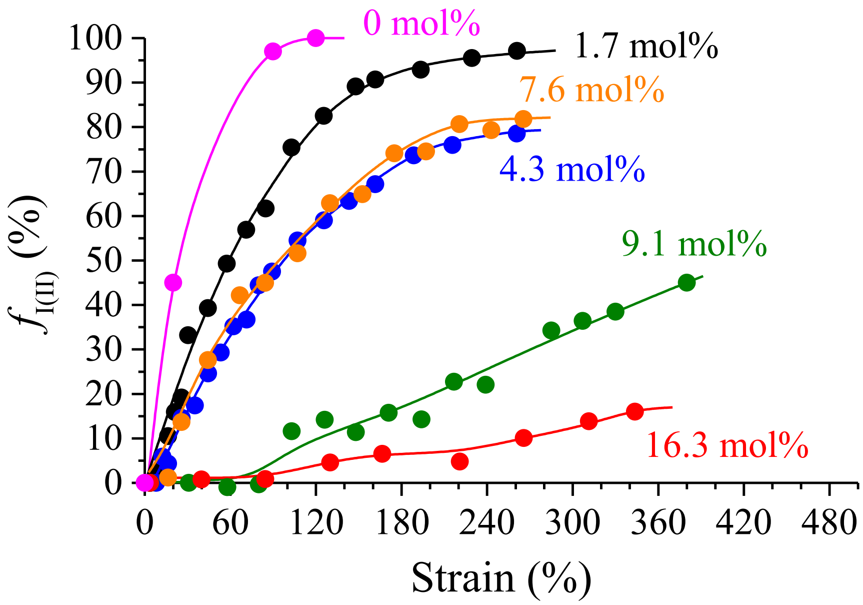

Figure S14. Percentage of form I obtained from the transition of form II ($f_{I(II)}$) for the iPB homopolymer (magenta), compared with the percentage measured for copolymers C4C2-1.7 (black), C4C2-4.3 (blue), C4C2-7.6 (orange), C4C2-9.1 (olive), and C4C2-16.3 (red), as a function of strain. The copolymers data are the same as those reported in Figure S12C. The ethylene content of each sample is indicated.

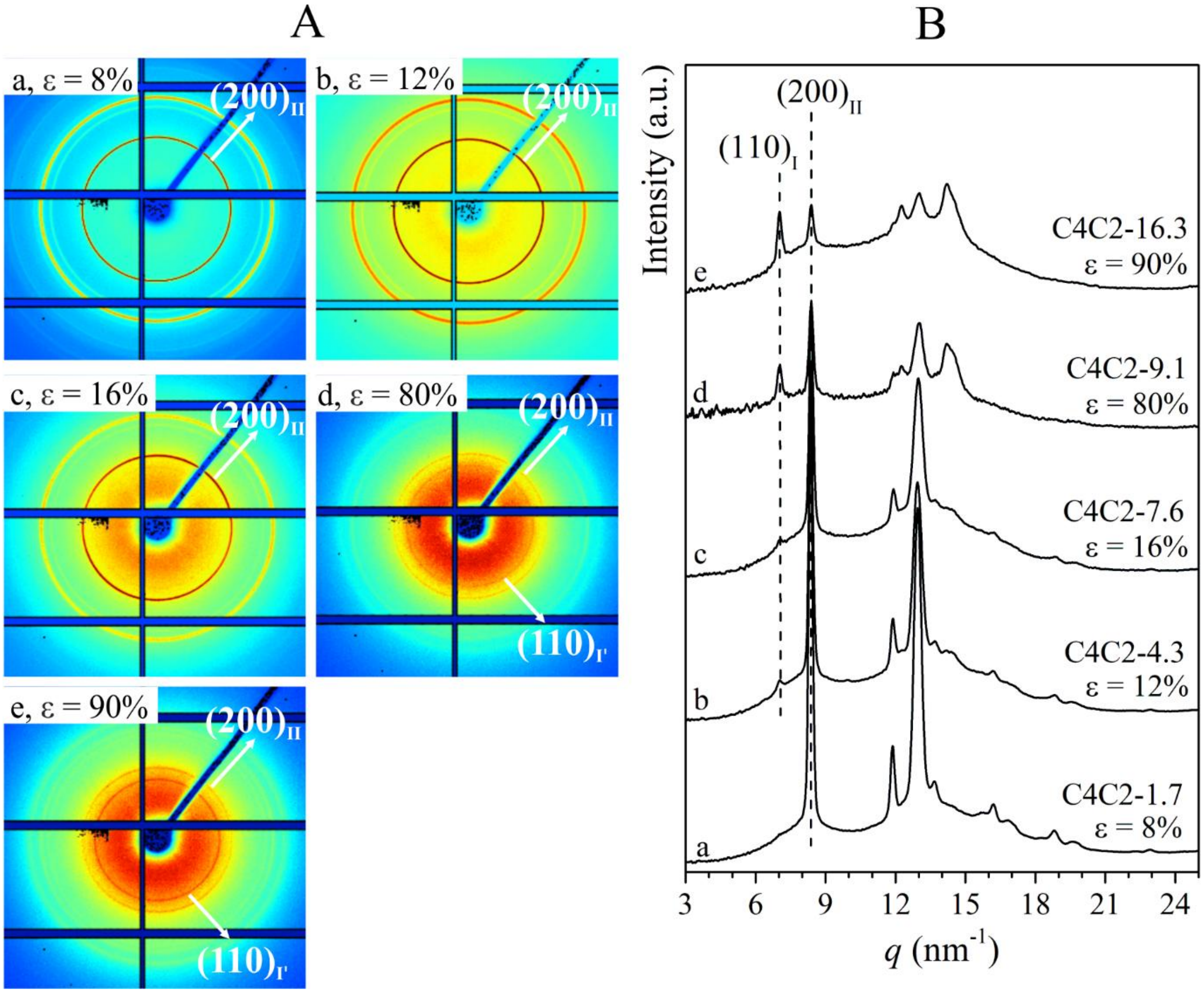

Figure S15. 2D WAXD patterns (A), and corresponding 1D diffraction profile integrated over the coordinate azimuthal χ (B), of the samples C4C2-1.7 (a), C4C2-4.3 (b), C4C2-7.6 (c), C4C2-9.1 (d) and C4C2-16.3 (e) recorded at the indicated values of strain ε, corresponding to critical strain at which the form II-form I transition begins (ε_c).

Calculation of the degree of crystalline orientation of form II during stretching

The degree of crystalline orientation of form II during stretching was quantified using Herman's orientation function f_H,[S7] calculated from the azimuthal intensity profiles $I(\chi)$ of the $(200)_{II}$ reflection of form II in the 2D WAXD patterns of the samples. In general, for uniaxially symmetric materials (such as fibers) the orientation function for a direction normal (pole) to any given {hkl} family of planes with respect to a preferred direction (fiber axis) is defined by eq. S6:

$$f_H = \frac{3\langle\cos^2\chi\rangle - 1}{2} \qquad (S6)$$

where $\langle\cos\chi\rangle$ is the average cosine of the angle χ that the poles make with the preferred direction and denotes the second order Legendre polynomial of argument $\langle\cos\chi\rangle$. The values of $\langle\cos\chi\rangle$, in turn, are calculated from the azimuthal intensity distribution of the *hkl* reflection as:

$$\langle\cos^2\chi\rangle = \frac{\int_0^\pi I(\chi)\cos^2\chi\sin\chi\, d\chi}{\int_0^\pi I(\chi)\sin\chi\, d\chi}$$

According to eq. S6, $f_H = 1$ corresponds to an ideal case of perfect alignment of the poles in the preferential direction, $f_H = 0$ corresponds to isotropic case and $f_H = -0.5$ corresponds to an ideal case of perfect perpendicular orientation.

In our case, $f_H = 1$ corresponds to a perpendicular orientation between the *c*-axis and the fiber axis (intensity concentrated along the meridian), $f_H = 0$ corresponds to the isotropic case (random orientation), and $f_H = -0.5$ represents a perfect parallel orientation of the c-axis to the fiber axis (intensity concentrated along the equator).

The Hermans' orientation functions (f_H) calculated from the azimuthal intensity profiles of the $(200)_{II}$ reflection of form II using eq. S6 for samples C4C2-1.7, C4C2-4.3, and C4C3-7.6, are reported in Figure S16 A, B and C, respectively, as a function of strain, together with the percentage of form I originating from the transition of form II ($f_{I(II)}$) during stretching. Figure S16D shows a comparison of the Hermans' orientation functions obtained for the three samples. The Hermans' orientation function (red data in Figure S16 A-C and Figure S11D) gradually decreases from 0 to negative values with increasing strain for all samples. This indicates that the crystallites of form II (initially isotropic) progressively tilt their chain axes with respect to the stretching direction, gradually approaching an equatorial orientation. Thus, as stretching proceeds, in parallel with the form II-to-form I transition, the residual form II crystallites progressively approach an equatorial orientation. This occurs in a similar way for all samples, reaching $f_H \approx -0.26$ at the maximum explored strain (Figure S16D).

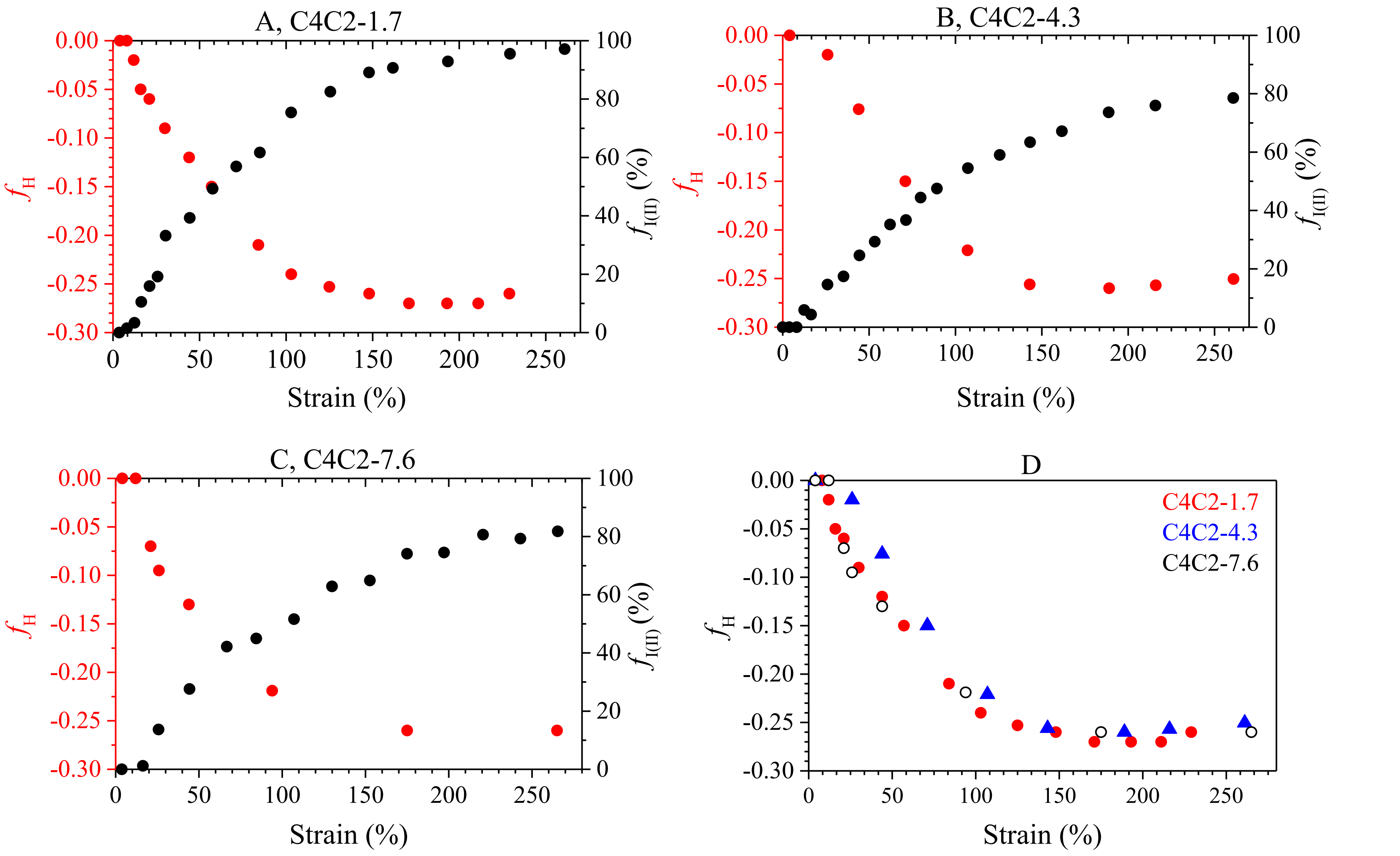

Figure S16. (A-C) Hermans' orientation function calculated from the azimuthal intensity profiles of the $(200)_{II}$ reflection of form II (f_H, red) and form I originated from transition of form II ($f_{I(II)}$, black) for samples C4C2-1.7 (A), C4C2-4.3 (B), and C4C2-7.6 (C), as a function of

strain. (D) Comparison of the Hermans' orientation functions obtained from the azimuthal intensity profiles of the $(200)_{II}$ reflection for the three samples: C4C2-1.7 (red circles), C4C2-4.3 (blue triangles), and C4C2-7.6 (black empty circles).

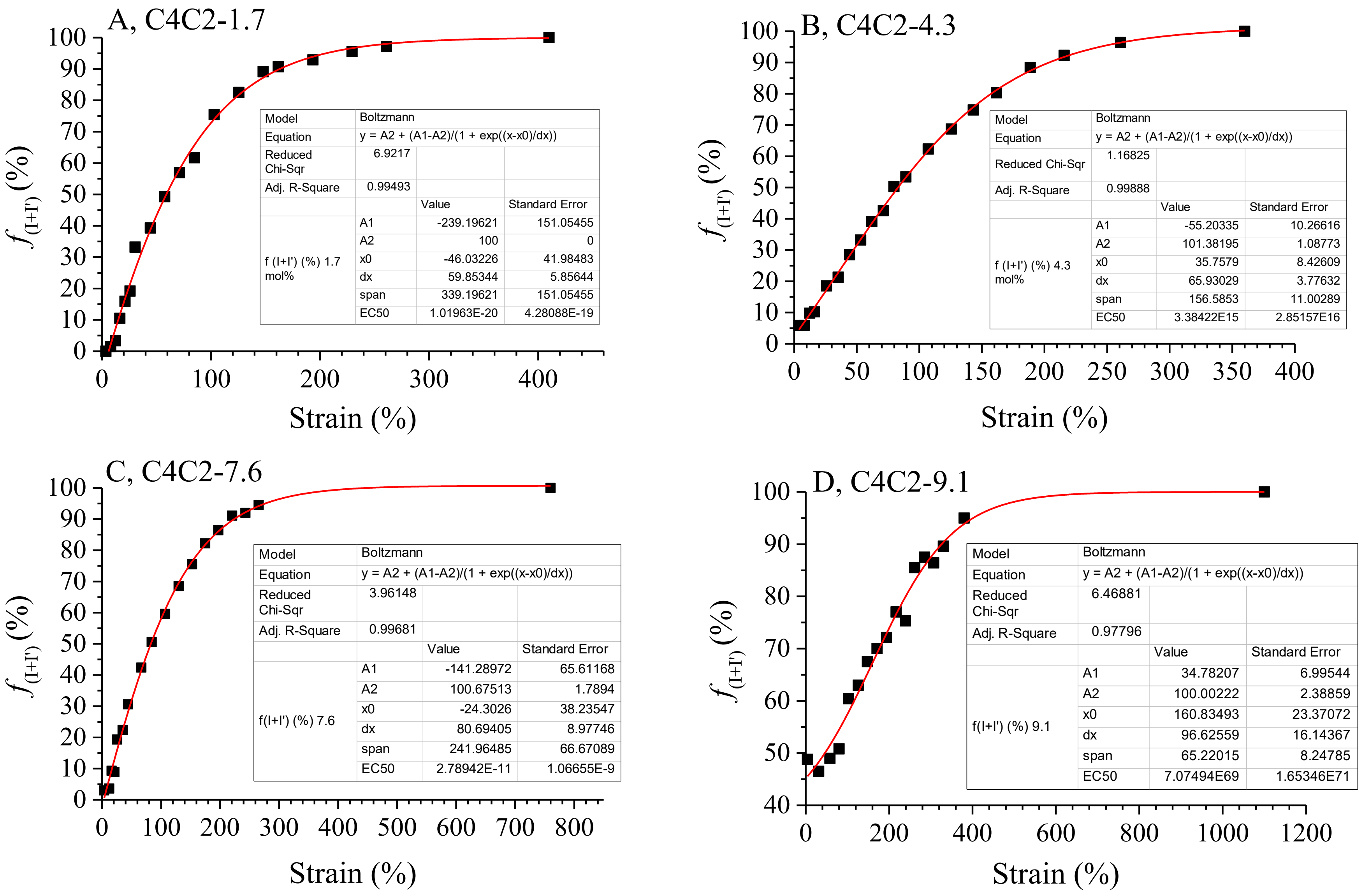
A, C4C2-1.7
$f_{(I+I')}$ (%)
Strain (%)
Model Boltzmann
Equation y = A2 + (A1-A2)/(1 + exp((x-x0)/dx))
Reduced Chi-Sqr 6.9217
Adj. R-Square 0.99493
Value Standard Error
f (I+I') (%) 1.7 mol%
A1 -239.19621 151.05455
A2 100 0
x0 -46.03226 41.98483
dx 59.85344 5.85644
span 339.19621 151.05455
EC50 1.01963E-20 4.28088E-19
B, C4C2-4.3
$f_{(I+I')}$ (%)
Strain (%)
Model Boltzmann
Equation y = A2 + (A1-A2)/(1 + exp((x-x0)/dx))
Reduced Chi-Sqr 1.16825
Adj. R-Square 0.99888
Value Standard Error
f (I+I') (%) 4.3 mol%
A1 -55.20335 10.26616
A2 101.38195 1.08773
x0 35.7579 8.42609
dx 65.93029 3.77632
span 156.5853 11.00289
EC50 3.38422E15 2.85157E16
C, C4C2-7.6
$f_{(I+I')}$ (%)
Strain (%)
Model Boltzmann
Equation y = A2 + (A1-A2)/(1 + exp((x-x0)/dx))
Reduced Chi-Sqr 3.96148
Adj. R-Square 0.99681
Value Standard Error
f(I+I') (%) 7.6
A1 -141.28972 65.61168
A2 100.67513 1.7894
x0 -24.3026 38.23547
dx 80.69405 8.97746
span 241.96485 66.67089
EC50 2.78942E-11 1.06655E-9
D, C4C2-9.1
$f_{(I+I')}$ (%)
Strain (%)
Model Boltzmann
Equation y = A2 + (A1-A2)/(1 + exp((x-x0)/dx))
Reduced Chi-Sqr 6.46881
Adj. R-Square 0.97796
Value Standard Error
f(I+I') (%) 9.1
A1 34.78207 6.99544
A2 100.00222 2.38859
x0 160.83493 23.37072
dx 96.62559 16.14367
span 65.22015 8.24785
EC50 7.07494E69 1.65346E71

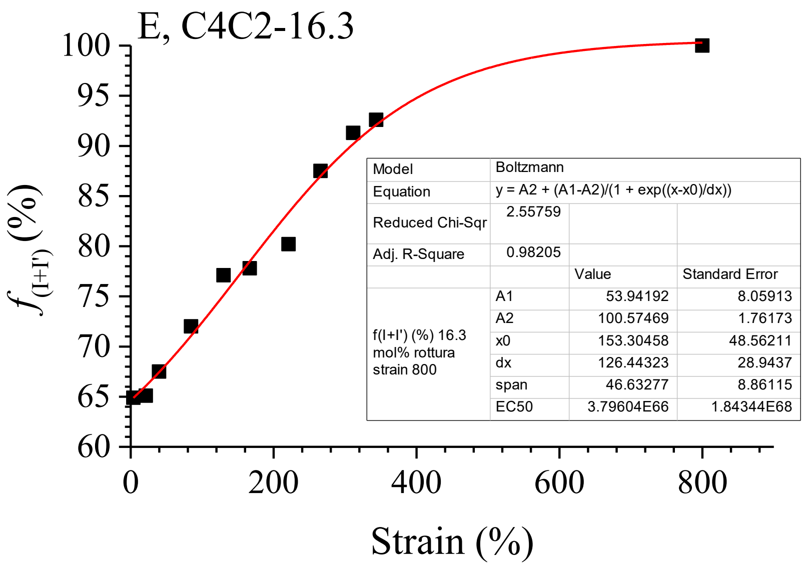

Figure S17. Fitted curves and extrapolation to 100% form I for the data reporting the percentage of global form I ($f_{(I+I')}$) as a function of strain (data from Figure S12B) for copolymers C4C2-1.7 (A), C4C2-4.3 (B), C4C2-7.6 (C), C4C2-9.1 (D), and C4C2-16.3 (E).

References

[S1] Sahoo, S. K.; Zhang, T.; Reddy, D. V.; Rinaldi, P. L. Multidimensional NMR Studies of Poly(ethylene-co-1-butene) Microstructures. *Macromolecules* **2003**, *36*, 4017-4028

[S2] Hsieh, E. T.; Randall, J. C. Ethylene-1-Butene Copolymers. 1. Comonomer Sequence Distribution. *Macromolecules* **1982,** *15*, 353-360.

[S3] De Rosa, C.; Auriemma, F.; Ruiz de Ballesteros, O.; Esposito, F.; Laguzza, D.; Di Girolamo, R.; Resconi, L. Crystallization Properties and Polymorphic Behavior of Isotactic Poly(1-Butene) from Metallocene Catalysts: The Crystallization of Form I from the Melt. *Macromolecules* **2009**, *42*, 8286-8297.

[S4] De Rosa, C.; Ruiz de Ballesteros, O.; Auriemma, F.; Di Girolamo, R.; Scarica, C.; Giusto, G. G.; Esposito, S.; Guidotti, S.; Camurati, I. Polymorphic Behavior and Mechanical Properties of Isotactic 1-Butene-Ethylene Copolymers from Metallocene Catalysts. *Macromolecules* **2014**, *47*, 4317–4329.

[S5] Ruiz de Ballesteros, O.; De Rosa, C.; Auriemma, F.; Malafronte, A.; Di Girolamo, R.; Scoti, M. Polymorphism and Form II–Form I Transformation in Ziegler–Natta Isotactic 1-Butene–Ethylene Copolymers Having a Multiblock Molecular Structure. *Polymer* **2020**, *198*, 122460.

[S6] Ruiz de Ballesteros, O.; Auriemma, F.; Di Girolamo, R.; Malafronte, A.; Scoti, M.; De Rosa, C. Mechanical properties of isotactic 1-butene-ethylene copolymers from Ziegler-Natta catalyst. *Polymer* **2021**, *216*, 123408.

[S7] Alexander, L.E. X-ray Diffraction Methods in Polymer Science; Wiley: New York, NY, USA, 1979.
